# Compound models and Pearson residuals for single-cell RNA-seq data without UMIs

**DOI:** 10.1186/s13059-026-04161-4

**Published:** 2026-06-27

**Authors:** Jan Lause, Christoph Ziegenhain, Leonard Hartmanis, Philipp Berens, Dmitry Kobak

**Affiliations:** 1 Hertie Institute for AI in Brain Health, University of Tübingen, Tübingen, Germany; 2 Tübingen AI Center, University of Tübingen, Tübingen, Germany; 3 Department of Medical Biochemistry & Biophysics, Karolinska Institutet, Stockholm, Sweden; 4 Department of Cell & Molecular Biology, Karolinska Institutet, Stockholm, Sweden; 5 VIB Center for AI and Computational Biology, VIB, Ghent, Belgium; 6 Department of Mathematics, Computer Science, and Statistics, Ghent University, Ghent, Belgium

## Abstract

Recent work employed Pearson residuals from Poisson or negative binomial models to normalize UMI-based scRNA-seq data. To extend this approach to non-UMI data, we model the amplification step with a compound distribution: we assume that captured RNA molecules follow a negative binomial distribution and are replicated following an amplification distribution. This model leads to compound Pearson residuals, yielding meaningful gene selection and embeddings of Smart-seq2 datasets. Furthermore, we show that amplification distributions across several sequencing protocols can be described by a broken power law. The resulting compound model captures previously unexplained overdispersion and zero-inflation patterns in non-UMI data.

**Supplementary Information:**

The online version contains supplementary material available at https://doi.org/10.1186/s13059-026-04161-4.

## Background

Single-cell RNA sequencing (scRNA-seq) data are affected by count noise and technical variability due to the total number of sequenced molecules varying from cell to cell. Removing this technical variation by normalization and variance stabilization is an important step in common analysis pipelines [1, 2]. The standard approach for this has been to use the log(1+x/s) transformation, where *s* is a size factor of the cell. While the log-transform often performs well in practice [3], it has well-known theoretical limitations and can produce biased results [4].

Recently, a number of count modelling approaches like sctransform [5], GLM-PCA [6], Sanity [7], and analytic Pearson residuals [8] have been suggested for pre-processing scRNA-seq data. These methods are based on explicit statistical models of the count generation process, rather than on heuristics such as the log-transform. One limitation of all of these methods is that they are tailored to data obtained using sequencing protocols based on unique molecular identifiers (UMIs), and are not appropriate for non-UMI technologies such as Smart-seq2 [9]. In this paper, we develop a count model and corresponding analytic Pearson residuals [8] for non-UMI sequencing data.

**Fig. 1:**
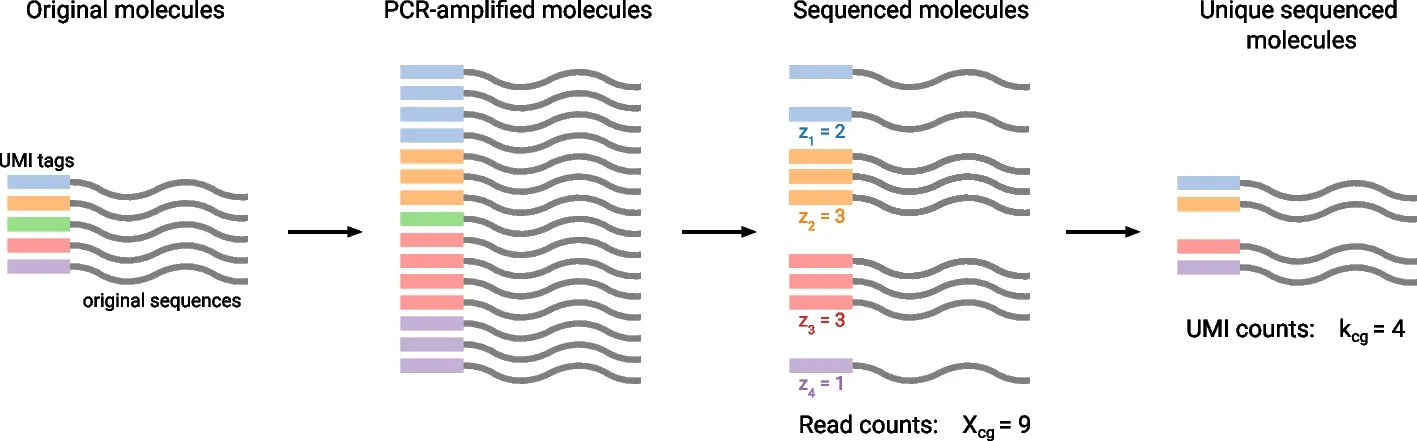
Important quantities in single-cell RNA sequencing. A cell *c* contains some number (here, 5) of RNA molecules of gene *g*. In UMI-based protocols, the original molecules are tagged by unique molecular identifiers (UMIs) before PCR amplification and sequencing. Both processes are imperfect, such that not all original molecules get amplified by the same factor, and not all amplified molecules get sequenced. In the end, each of the unique molecules may get sequenced one or several times, which is its *copy number* ($z_i$). Here $z_i$ values are 2, 3, 3, and 1, and one original molecule (green) is not detected at all. The sum of all copy numbers gives the observed *read count* $X_{cg}$ (here, 9). UMIs allow to compute a *UMI count* $k_{cg}$ by only counting unique sequenced molecules (here, 4). In non-UMI protocols, amplification and sequencing work the same, but the final de-duplication step is not possible, meaning that only $X_{cg}$ is observable. Note that the shown numbers are not to scale: A typical cell might contain on the order of $10^5$ RNA molecules across all genes [10]; yielding on the order of $10^{10}$ molecules after amplification; and producing on the order of $10^6$ sequenced reads

Single-cell sequencing protocols usually require an amplification step by polymerase chain reaction (PCR) to obtain enough starting material for sequencing (Fig. 1). The process is imperfect, and different molecules will get amplified to a different extent. As a result, the number of sequenced molecules of a given gene (called *read count*) does not reflect the original number of RNA molecules in the cell.

In UMI protocols, a random DNA sequence called UMI is appended to each original reverse-transcribed RNA molecule prior to the amplification, and is then amplified and sequenced together with it [11]. Because the UMI uniquely identifies each original molecule, one can later remove amplification duplicates by counting each UMI only once (‘de-duplication’), giving rise to the *UMI counts* instead of *read counts* (Fig. 1), effectively reducing amplification noise [12]. Note that the UMI count does not necessarily equal the number of original RNA molecules present in the cell, as some molecules can get lost during sample preparation for the sequencing (‘library preparation’) or fail to get sequenced due to low capture rate (‘depth’) in the sequencing step (Fig. 1).

While UMI protocols are popular in the scRNA-seq community, non-UMI technologies remain important. Indeed, UMIs only mark one end of the original molecules, so UMI counts are not available for the internal reads (most protocols involve a fragmentation step that cuts each molecule into small fragments prior to sequencing). Full-length sequencing methods, such as Smart-seq2 [9], are typically more sensitive than UMI protocols [13, 14], and are often used to detect rare cell types [15, 16] or splicing variants [17], or in low-throughput experiments such as Patch-seq [18]. In the resulting datasets, UMI counts are not available, and computational analysis has to be based on the read counts that still contain amplification-induced variability.

Only few normalization methods have been developed specifically to account for the amplification noise in read counts. The *Census* [19] and *quasi-UMIs* (qUMIs) [20] methods are two transformations that are designed to make the shape of the read-count distribution approximately match the shape of the UMI count distribution. Afterwards, the transformed data still requires a UMI-like normalization. However, neither Census nor qUMIs derive their transforms from a principled statistical model and rather rely on heuristics.

Here, we develop a new theoretically motivated method for normalization of non-UMI data that explicitly accounts for the amplification noise: *compound Pearson residuals*. We do so by extending the null model behind the analytic Pearson residuals [8] from UMI counts to read counts, based on an explicit statistical model for the amplification step. This yields a generative model for read counts that reproduces characteristic patterns of non-UMI data. We demonstrate that our compound Pearson residuals can efficiently normalize complex read-count datasets.

## Results

### Analytic Pearson residuals for normalization of UMI data

In this section we briefly summarize the normalization approach based on Pearson residuals, originally developed by Hafemeister and Satija [5] for UMI data. Pearson residuals compare the observed data to a null model that captures only technical variability due to count noise and variations in sequencing depth. The null model assumes perfect biological homogeneity, and so any deviation from it suggests biological variability.

Under the null model [8], a gene *g* takes up a certain constant fraction $p_g$ of the total $n_c$ RNA molecules sequenced in cell *c*, and the observed UMI counts $k_{cg}$ follow a negative binomial (NB) distribution:

1 $$\begin{aligned} k_{cg} \sim \mathrm{NB}(\mu _{cg}, \theta ),\end{aligned}$$

2 $$\begin{aligned} \mu _{cg} = n_c p_g, \end{aligned}$$

where θ is the inverse overdispersion parameter. Higher values of θ yield smaller variance, and for $\theta =\infty$, the NB distribution reduces to the Poisson distribution. Note that θ in this formulation is shared between all genes; based on negative control UMI data, we previously argued [8] that θ can be set to θ=100, which is close to Poisson. Others have suggested pure Poisson as measurement model [21].

Given an observed UMI count matrix, the maximum likelihood estimate of $\mu _{cg}$ is given by:

3 $$\begin{aligned} \hat{\mu }_{cg} = \frac{\sum \nolimits _j k_{cj} \cdot \sum \nolimits _i k_{ig} }{\sum \nolimits _{ij} k_{ij}}, \end{aligned}$$

which is exact in the Poisson case and holds only approximately in the NB case [8]. This yields the analytic formula for *UMI Pearson residuals* (difference between observed UMI count values and model prediction, divided by the model standard deviation):

4 $$\begin{aligned} R^{\textrm{UMI}}_{cg} = \frac{k_{cg} - \hat{\mu }_{cg}}{\sqrt{\hat{\mu }_{cg}+\hat{\mu }_{cg}^2/\theta }}, \end{aligned}$$

where $\hat{\mu }_{cg}+\hat{\mu }_{cg}^2/\theta$ is the variance of the NB distribution with mean $\hat{\mu }_{cg}$ and overdispersion parameter θ. The variance of Pearson residuals does not depend on $p_g$, and in a homogeneous dataset is close to 1 for all genes. This ensures variance stabilization across all levels of gene expression.

This algorithm is similar to the one implemented in sctransform [5, 22] and is equivalent to a rank-one GLM-PCA [6], but it is simpler and faster to compute than either of these methods. When followed by singular value decomposition (SVD), the Poisson ($\theta =\infty$) version of UMI Pearson residuals is also known as correspondence analysis [23].

### Compound Pearson residuals for non-UMI read-count data

To apply Pearson residuals to scRNA-seq data without UMIs, we need to change the null model, because read counts do not follow the NB distribution [24, 25]. As in the UMI case, we assume that the number of unique sequenced RNA molecules $k_{cg}$ follows a Poisson or a NB distribution. However, during the amplification step, each of these $k_{cg}$ unique molecules could have been duplicated multiple times before sequencing (Fig. 1). For the *i*-th unique molecule, we call the number of its sequenced duplicates its *copy number* $z_i$. We assume that copy numbers follow some distribution Z, which we call *amplification distribution*. Our assumption is that the amplification distribution is the same for all genes and all cells, and only depends on the details of the PCR amplification and the sequencing protocol (see the note below about the variable gene length).

The read count $X_{cg}$ of a given gene *g* in cell *c* is thus modeled as the sum of $k_{cg}$ independent and identically distributed (i.i.d.) positive integer copy numbers drawn from Z:

5 $$\begin{aligned} X_{cg} = \sum \limits _{i=1}^{k_{cg}}{z_i}, \end{aligned}$$

6 $$\begin{aligned} z_i \sim \mathrm Z\text { with } z_i \in \mathbb {N}^+, \end{aligned}$$

7 $$\begin{aligned} k_{cg} \sim \mathrm{NB}(\mu _{cg}, \theta ), \end{aligned}$$

8 $$\begin{aligned} \mu _{cg} = n_c p_g. \end{aligned}$$

For example, $k_{cg}=4$ means that four unique RNA molecules of gene *g* were sequenced in a cell *c*; if their copy numbers were 2, 3, 3, and 1, this would yield the read-count value $X_{cg} = 2+3+3+1 = 9$ (c.f. Fig. 1). The resulting distribution of $X_{cg}$ can be called *compound NB distribution* (see Methods).

In the above formulation, our model does not explicitly account for gene length. In most sequencing protocols, longer transcripts are cut into more fragments before amplification. This will result in more unique sequenced fragments $k_{cg}$ for longer molecules [26]. In our model, this increase amounts to a constant length factor $l_g$ per gene, which can be absorbed by our per-gene expression fraction $p_g$. The variance of Pearson residuals does not depend on $p_g$ (see above), so for simplicity, we do not explicitly put gene lengths into the model.

To obtain Pearson residuals for this null model, we need to obtain expressions for its mean and variance. The mean of a compound NB distribution is equal to the product of the NB mean $\mu _{cg}$ and the mean of the amplification distribution Z:

9 $$\begin{aligned} \mathbb {E}[X_{cg}] = \mathbb {E}[Z] \cdot \mathbb {E}[k_{cg}] = \mathbb {E}[Z] \cdot \mu _{cg}. \end{aligned}$$

We can use the observed read-count matrix *X* to estimate the means of the maximum likely null compound model as follows:

10 $$\begin{aligned} \hat{\mathbb {E}}[X_{cg}] \approx \mathbb {E}[Z] \cdot \hat{\mu }_{cg} = \frac{\mathbb {E}[Z]^2}{\mathbb {E}[Z]} \cdot \frac{\sum \nolimits _j k_{cj} \cdot \sum \nolimits _i k_{ig} }{\sum \nolimits _{ij} k_{ij}} \approx \frac{\sum \nolimits _j X_{cj} \cdot \sum \nolimits _i X_{ig} }{\sum \nolimits _{ij} X_{ij}}. \end{aligned}$$

Note that this expression has the same form as Eq. 3: the outer product of the row and the column sums of the count matrix, normalized by its total sum.

The mean-variance relationship of the compound NB distribution takes the form (see Methods):

11 $$\begin{aligned} \text {Var}[X_{cg}] = \alpha _Z \mathbb {E}[X_{cg}] + \frac{\mathbb {E}[X_{cg}]^2}{\theta },\end{aligned}$$

12 $$\begin{aligned} \,\,\textrm{where}\,\, \alpha _Z = \mathbb {E}[Z] + \mathbb{F}\mathbb{F}[Z] = \mathbb {E}[Z] + \frac{\text {Var}[Z]}{\mathbb {E}[Z]}. \end{aligned}$$

This expression is similar to the mean-variance relationship of the NB distribution but contains a scaling parameter $\alpha _Z$ equal to the sum of the mean and the Fano factor (denoted $\mathbb{F}\mathbb{F}[Z]$) of Z. Note that in the compound Poisson case ($\theta =\infty$), the compound variance is proportional to the compound mean.

Using these equations, we can compute the Pearson residuals of the compound NB null model, which we call the *compound Pearson residuals*:

13 $$\begin{aligned} R_{cg} = \frac{X_{cg}-\hat{\mathbb {E}}[X_{cg}]}{\sqrt{ \alpha _Z \hat{\mathbb {E}}[X_{cg}] + \hat{\mathbb {E}}[X_{cg}]^2 / \theta }}. \end{aligned}$$

Following the UMI case [8], we set the overdispersion parameter to θ=100. The scalar $\alpha _Z$ is a function of the mean and variance of the amplification distribution and remains as the only free parameter of the model. We clip the residuals to $[-\sqrt{n},\sqrt{n}]$, where *n* is the number of cells in the dataset [5, 8].

This formalism naturally generalizes the UMI Pearson residuals. Indeed, in the UMI case, each sequenced molecule is counted only once, thanks to the UMIs, meaning that the Z distribution is a delta peak δ(1) with $\mathbb {E}[Z]=1$ and $\text {Var}[Z]=0$, and hence $\alpha _Z=1$. In this case, Eq. 13 reduces to Eq. 4.

Conveniently, compound Pearson residuals are equivalent to UMI Pearson residuals of the read-count matrix scaled by $1/\alpha _Z$:

14 $$\begin{aligned} R_{cg}(X_{cg}; \alpha _Z, \theta ) = \frac{(X_{cg}-\hat{\mathbb {E}}[X_{cg}])/\alpha _Z}{\sqrt{\left( \alpha _Z \hat{\mathbb {E}}[X_{cg}] + \hat{\mathbb {E}}[X_{cg}]^2 / \theta \right) /\alpha _Z^2}} = R^{\textrm{UMI}}_{cg}(X_{cg}/\alpha _Z; \theta ). \end{aligned}$$

Importantly, the necessary scaling factor is not equal to E[Z], as could be naïvely expected, but rather to $\alpha _Z = \mathbb E[Z] +\text {Var}[Z]/\mathbb {E}[Z]$.

Compound Pearson residuals have the same computational complexity as UMI Pearson residuals, and can be computed in seconds even for large datasets with ${>}10\,000$ cells. The matrix of Pearson residuals is dense and will thus require more memory than the sparse matrix of raw counts. For very large datasets it may be prohibitive to hold the full matrix in memory, but this can be avoided by directly performing principal component analysis (PCA) on the residuals [27] or by subsetting to highly variable genes [8], allowing to process datasets with millions of cells.

### Compound model can fit homogeneous read-count data

**Fig. 2:**
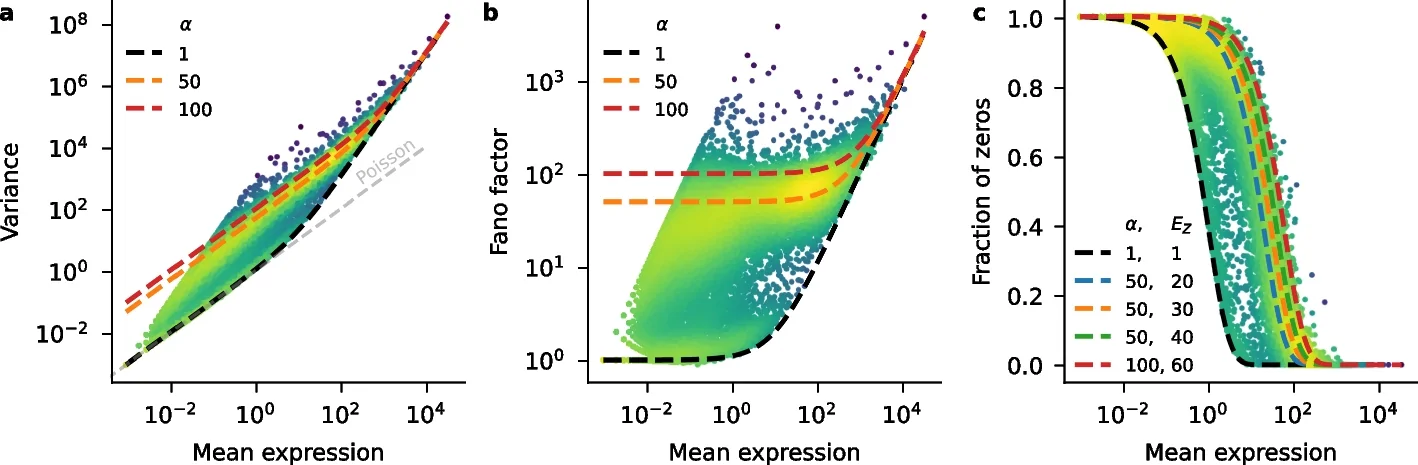
The compound NB model captures statistics of homogeneous read-count data. The data are a homogeneous subset of a mouse visual cortex dataset [15] sequenced with Smart-seq2 (*L6 IT VISp Penk Col27a1* cluster; 1049 cells, 33914 genes). Each dot represents a gene. Brighter colors indicate higher density of points. Dashed lines show the behavior of the compound negative binomial model (θ=10). **a** Mean-variance relationship. Gray line illustrates the Poisson case where mean equals variance. **b** Relationship between mean expression and Fano factor (variance/mean). **c** Relationship between mean expression and fraction of zero counts

The compound model introduced above is designed to capture only technical, but not biological variance in non-UMI read-count data. Therefore, it should provide a good fit to data that contain little biological variation. To test this, we took scRNA-seq data from adult mouse neocortex sequenced with Smart-seq2 [15], and focused on a subset of cells corresponding to one specific cell type, assuming that there is little biological variability within a cell type. We chose the *L6 IT VISp Penk Col27a1* type (as annotated by the authors of the original study), containing 1049 excitatory neurons (Fig. 2).

The mean-variance relationship across genes (Fig. 2a) showed that most genes exhibited overdispersion compared to the Poisson model (gray line, $\alpha _Z=1$ and $\theta =\infty$). Most genes also showed more variance than expected from a NB model without amplification (black line, $\alpha _Z=1$ and θ=10). In contrast, compound models accounting for amplification with $\alpha _Z\gg 1$ (colored lines) were able to approximate the mean-variance relationship for the majority of the genes. Note that we used θ=10 for illustrations in Fig. 2 because this value fit the within-cell-type data better than θ=100, in agreement with the idea that even biologically homogeneous data can show some additional variability on top of the purely technical variability [8]. Cell-to-cell variation in sequencing depth also contributed to this increase in overdispersion (Additional File 1: Supplementary Figure S1).

The relationship between the mean and the Fano factor across genes (Fig. 2b) was bounded by a model with $\alpha _Z=1$ from below (black), and by a model with $\alpha _Z=100$ from above (red). The bulk of the genes followed a model with $\alpha _Z \approx 50$ (orange). The observed Fano factors can be used directly to estimate the amplification parameter $\alpha _Z$, because in our model, for genes with low average expression, the Fano factor of the read counts is equal to $\alpha _Z$ (Eqs. 11 and 29 in the Methods). Based on this relationship, we estimated $\alpha _Z=57$ as the mode of the distribution of Fano factors (Additional File 1: Supplementary Figure S2a).

While knowing $\alpha _Z$ is sufficient to compute Pearson residuals, we can obtain separate estimates of $\mathbb {E}[Z]$ and $\text {Var}[Z]$ by studying the relationship between the average expression and the fraction of zeros. This relationship only depends on $\mathbb {E}[Z]$ (see Methods, Eq. 30), allowing to estimate this term directly. We observed that in the Smart-seq2 data, the fraction of observed zeros decreased with increasing mean expression (Fig. 2c). There were more observed zeros than expected from a NB model with $\alpha _Z=1$ (black), hinting at why read-count data have in the past often been modeled using a zero-inflated negative binomial (ZINB) distribution (e.g., [28, 29]). We could estimate the value of $\mathbb {E}[Z]$ directly from this data using a linear fit (Eq. 32), obtaining $\mathbb {E}[Z]=36$ (Additional File 1: Supplementary Figure S2b). From here we can compute $\mathbb{F}\mathbb{F}[Z]=\alpha _Z-\mathbb {E}[Z]=57-36=21$ and hence $\text {Var}[Z]\approx 21\cdot 36\approx 750$.

The compound NB model with $\alpha _Z=50$ and $\mathbb {E}[Z]=30$ described the majority of genes well. However, some genes were instead following the model without any amplification (black line in Fig. 2), as if their transcripts were not amplified by the PCR. To understand this pattern, we obtained gene type annotations from mygene.info. This revealed that protein-coding genes generally followed our best-fitting compound model (Additional File 1: Supplementary Figure S3a–c), while most of the seemingly non-amplified genes were pseudogenes (Additional File 1: Supplementary Figure S3d–f). This observation was not limited to the Smart-seq2 data, but also occurred for various other sequencing protocols (Additional File 1: Supplementary Figure S4). Exonic transcript lengths from the mygene.info database were shorter for the non-amplified genes (Additional File 1: Supplementary Figure S5).

In summary, we showed that our compound NB model fits a biologically homogeneous example dataset. In particular, our model matched the main statistical properties of protein-coding genes (mean, variance, and fraction of zeros). Furthermore, there is a simple way to estimate the model parameters $\alpha _Z$ and $\mathbb {E}[Z]$ (which we will validate using simulations in the next section). When we applied this estimation procedure to the entire mouse neocortex dataset [15], and not only to a single cell type as above, we obtained $\alpha _Z=117$ and E[Z]=57 (Additional File 1: Supplementary Figure S2c–d). This is because of the differences in observed amplification statistics between cell types: in Smart-seq2, all cell types get roughly the same number of sequencing reads (because the amount of amplified cDNA to be sequenced is set by the experimenter), so cell types with fewer expressed genes (e.g., non-neuronal cells) get more reads per molecule (Additional File 1: Supplementary Figure S6). In the following text, we will use $\alpha _Z=50$ as the default value for analysis.

### Compound Pearson residuals recover ground-truth marker genes and cell types

In this section, we used simulations to test the ability of compound Pearson residuals to preserve biologically relevant signals for downstream analysis. We based our simulations on the full mouse neocortex dataset [15], which is highly heterogeneous and includes both neural and non-neural cells from two areas of the mouse neocortex. Specifically, we took sequencing depths $n_s$, gene fractions $p_g$, and cell-type identities from the real data, and then used a compound model with $\textrm{NB}(\theta =100)$ as UMI distribution and a geometric distribution with mean 100 as amplification distribution to simulate counts within each simulated cell type. The true $\alpha _Z$ in this case is approximately 200 (see Methods).

We then compared the ability of compound Pearson residuals, UMI Pearson residuals, qUMI [6], and Census [19] to recover the ground-truth marker genes and the ground-truth cell types from the simulated data. In order to demonstrate the robustness of compound Pearson residuals, we used $\alpha _Z=50$ to compute them, which was not the same value as actually used in the simulation. For HVG selection with Pearson residuals, we used the variance of residuals for each gene. Census and qUMI estimate non-normalized UMI counts, so we processed them with standard Scanpy [30] functions for normalization and HVG selection at their default settings (this includes depth normalization, log1p() transform, and “Seurat” HVG selection). For all methods, we used 3000 HVGs and reduced them to 1000 principal components.

**Fig. 3:**
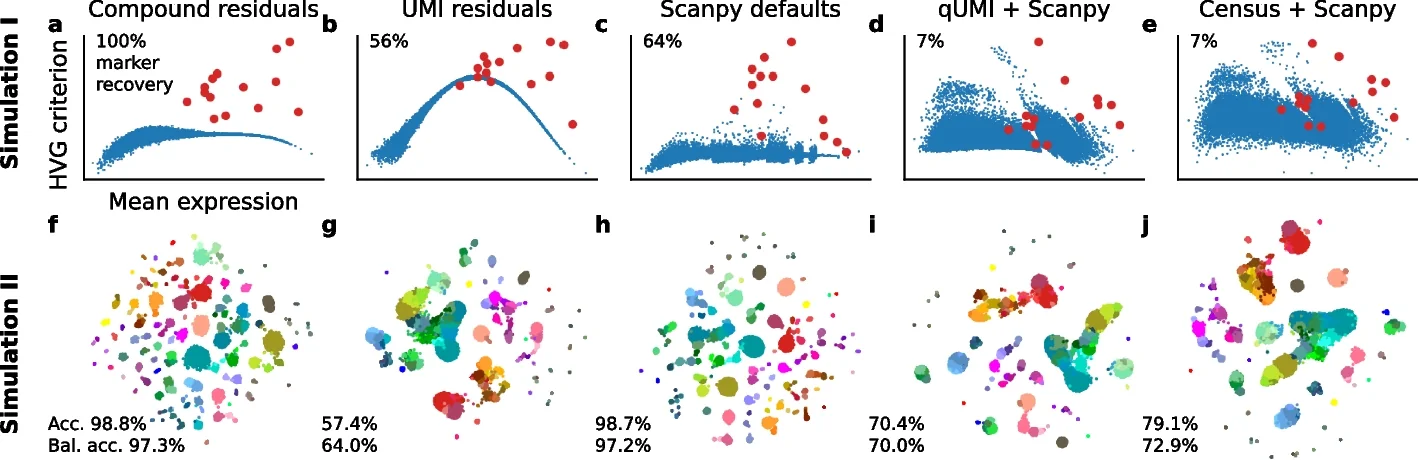
Compound Pearson residuals recover ground truth in simulated data. **a**–**e** *Simulation I*, in which the expression of only 14 genes varied between simulated cell types. The panels show the relationship between the log-scaled mean expression of the simulated read counts and the HVG selection criterion of the respective method (residual variance on a log scale in (**a**)–(**b**) and normalized dispersion on a linear scale in (**c**)–(**e**)). Each dot is one of 21321 simulated genes; red dots correspond to simulated marker genes. **f**–**j** *Simulation II*, in which the expression of all 21397 simulated genes was allowed to vary between simulated cell types. The panels show *t*-SNE embeddings of the data after the respective transformation, followed by HVG selection and PCA. Each dot is a cell, colored by simulated cell type. Numbers in each panel denote the *k*NN accuracy (top) and the balanced *k*NN accuracy (bottom) for cell type prediction in PCA space

In *Simulation I*, we focused on marker gene recovery. We allowed only a small set of 14 well-known marker genes specific for cortical cell types (*Itgam, Bgn, Pdgfra, Aqp4, Flt1, Foxp2, Mog, Rorb, Pvalb, Slc17a7, Gad1, Sst, Vip, Snap25*) to vary between cell types and set the expression of all other genes to be the same for all cell types. A successful HVG selection procedure should rank these 14 ground-truth marker genes first. Compound Pearson residuals indeed perfectly recovered all of them (Fig. 3a). In contrast, UMI Pearson residuals ($\alpha _Z=1$) showed high residual variance for many non-variable genes (56% marker gene recovery, Fig. 3b). Applying Scanpy defaults directly to read counts recovered 64% of marker genes (Fig. 3c), while qUMI and Census followed by Scanpy defaults only achieved 7% (Fig. 3d–e) and mostly selected non-variable genes as HVGs.

In *Simulation II*, we focused on cell-type recovery. Here we used cell-type-specific $p_g$ values for each gene, thereby creating 133 ground-truth cell types (classes) with the same mean expression as cell types in the real data. To quantify cell-type preservation, we computed the *k*NN accuracy (k=10) after HVG selection and PCA, i.e., the fraction of cells for which the majority of their 10 nearest neighbors has the same cell type. Compound Pearson residuals achieved 98.8% accuracy and 97.3% balanced accuracy (average per-class accuracy, subsequently averaged over classes) (Fig. 3f). Scanpy performed around the same (98.7% and 97.2% respectively) (Fig. 3h), but UMI residuals, qUMI, and Census all yielded much lower *k*NN accuracy values (57.4%, 70.4%, 79.1%; Fig. 3g,i,j). These numeric results agree with the qualitative appearance of the respective *t*-SNE embeddings.

Additionally, *Simulation II* allowed us to validate the parameter estimation procedure introduced in the previous section. When applied to the simulated dataset, it estimated $\alpha _Z = 202$ and $\mathbb {E}[Z] = 104$ (Additional File 1: Supplementary Figure S7a–b) using cells from a single cluster, very close to the true values. When using cells from the entire datasets, the estimates were almost the same (Additional File 1: Supplementary Figure S7c–d).

In summary, both simulation experiments confirmed that compound Pearson residuals can recover true marker genes and true cell types, and validated our parameter estimation procedure. Importantly, UMI residuals did not perform well in these simulations, highlighting why compound residuals are needed for processing read counts. Compound Pearson residuals also outperformed both qUMI and Census.

### Compound Pearson residuals for normalization of heterogeneous read-count data

**Fig. 4:**
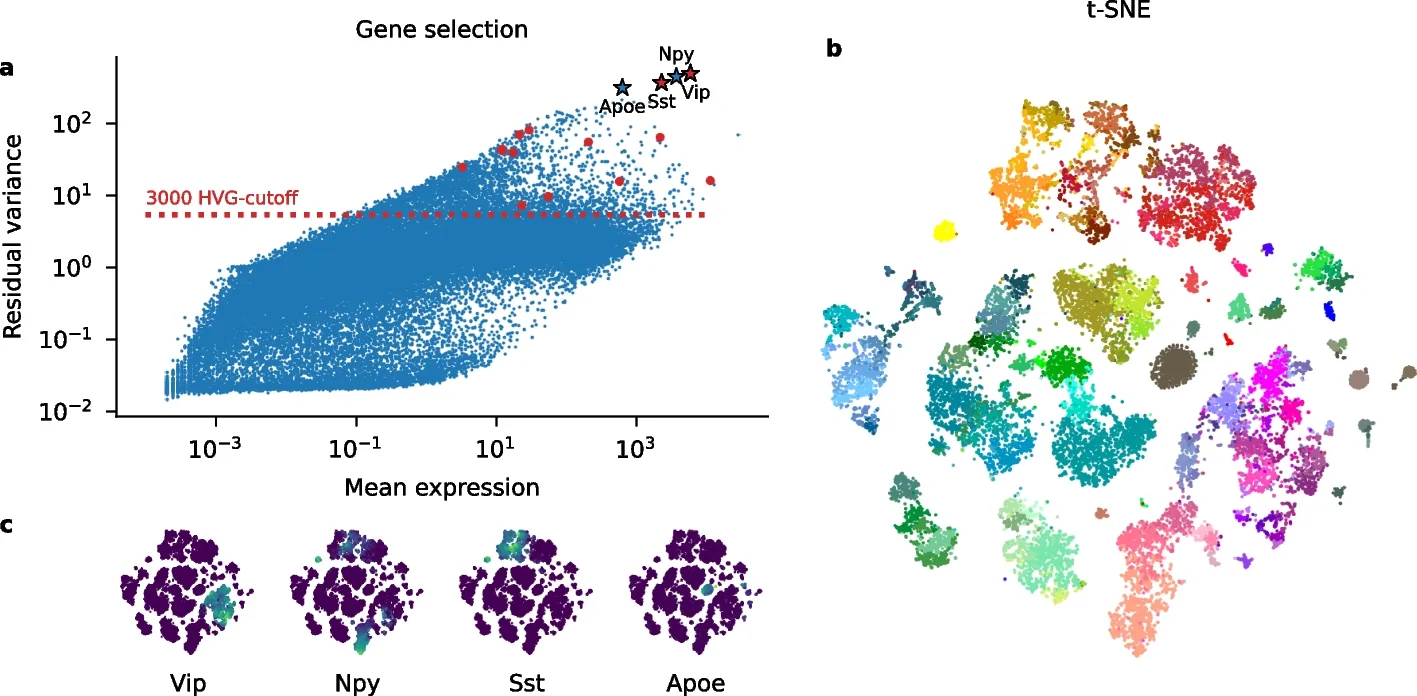
Compound Pearson residuals work well for preprocessing a heterogeneous Smart-seq2 dataset. We used the raw counts of a mouse visual cortex dataset [15] (23822 cells, 38510 genes). We used a compound NB model with amplification parameter $\alpha _Z=50$ and overdispersion parameter θ=100. **a** Highly variable gene (HVG) selection by largest residual variance. Each dot is a gene; genes above the red line were included in the selection of 3000 HVGs. Stars indicate the top four HVGs shown in panel (**c**). Red dots and stars correspond to the following well-known marker genes [31], from left to right: *Itgam* (microglia), *Bgn* (smooth muscle cells), *Pdgfra* (oligodendrocyte precursors), *Aqp4* (astrocytes), *Flt1* (endothelial cells), *Foxp2* (layer 6 excitatory neurons), *Mog* (oligodendrocytes), *Rorb* (layer 4 excitatory neurons), *Pvalb* (subset of inhibitory neurons), *Slc17a7* (excitatory neurons), *Gad1* (inhibitory neurons), *Sst* (subset of inhibitory neurons), *Vip* (subset of inhibitory neurons), *Snap25* (neurons). **b** *t*-SNE embedding on compound Pearson residuals following HVG selection (to 3000 HVGs) and PCA (down to 1000 PCs). Each dot is a cell, colored using the cluster colors from the original publication. Warm colors: inhibitory neurons. Cold colors: excitatory neurons. Brown and gray colors: non-neural cells. **c** *t*-SNE embeddings as in panel (**b**), colored by expression strength of the four most variable genes according to compound Pearson residual variance. For the colormap we used square-root-transformed, depth-normalized counts

In this section, we apply our method to the full mouse neocortex dataset [15]. For that, we used compound Pearson residuals with $\alpha _Z=50$ (Eq. 13) to process the full dataset with the same procedure we described in the previous section. The mean-variance relationship after normalization (Fig. 4a) showed that most genes had residual variance close to 1, i.e., they followed the null model. The interpretation is that most genes did not show biological variability and were not differentially expressed between cell types. In contrast, genes with residual variance ≫1 had more variability than predicted by the null model, implying nontrivial biological variability. For downstream analysis, we again selected the 3000 HVGs with highest residual variances. Among those were multiple well-known marker genes (Fig. 4a, red dots). In particular, the four genes with the highest residual variance (star symbols in Fig. 4a) marked different groups of inhibitory neurons (*Npy*, *Vip*, *Sst*) and astrocytes (*Apoe*). As before, we confirmed that genes with very low residual variances ≪1 were mostly pseudogenes (Additional File 1: Supplementary Figure S8).

Next, we used PCA and *t*-SNE to visualize the single-cell composition of the mouse cortex using compound Pearson residuals of the selected HVGs. The resulting embedding showed rich structure that corresponded well to the cell-type annotations determined in the original publication (Fig. 4b): individual cell types formed mostly clearly delineated clusters, while related cell types (having similar colors) mostly stayed close to each other. The expression of most variable genes according to the residual variance was typically localized in one part of the embedding space (Fig. 4c).

Calculating compound Pearson residuals requires to set the amplification parameter $\alpha _Z$. As we showed before, we can estimate this parameter either from one homogeneous neuronal cluster as $\alpha _Z=57$ or from the full dataset as $\alpha _Z=117$ because non-neuronal cell types with less initial RNA get higher copy numbers (Additional File 1: Supplementary Figure S6). For simplicity, we used α=50 as the default value for all analyses, but the results with $\alpha _Z=100$ were qualitatively almost identical (Additional File 1: Supplementary Figure S9). At the same time, using UMI Pearson residuals with $\alpha _Z=1$ yielded visibly worse results (Additional File 1: Supplementary Figure S9a–b), showing again that it is not appropriate to apply UMI Pearson residuals to non-UMI data. This matches our simulation-based comparisons, where UMI residuals performed worse than compound residuals.

We compared the performance of our approach with existing methods: UMI Pearson residuals (i.e., α=1), Scanpy defaults, and qUMI and Census followed by Scanpy. We argue that it is not possible to use cell-type classification for quantitative comparisons here because of the lack of ground-truth cell-type labels (which is why above we used simulations for benchmarking). Still, we found that compound Pearson residuals captured a set of well-known marker genes better than other methods (Additional File 1: Supplementary Figure S10).

As noted above, computing compound Pearson residuals was fast. For this dataset with ca. 23000 cells and 38000 genes it took ∼15 s on a single CPU. The resulting dense matrix of residuals used 3.4 Gb of RAM instead of 1.6 Gb for the sparse matrix of read counts. Census and qUMI had much slower runtimes (3 h and 3 min respectively). For a more systematic runtime comparison, we subsampled/upsampled the mouse neocortex dataset [15] to sample sizes from 1 000 to 100 000 (Additional File 1: Supplementary Table S1). Compound Pearson residuals had only a minor runtime overhead over the standard Scanpy preprocessing.

### The broken zeta distribution as amplification model

So far, we did not specify the amplification distribution Z. Instead, we only characterized its mean and variance through the $\alpha _Z$ parameter. While this was sufficient to compute compound Pearson residuals, an explicit amplification distribution is needed for the complete specification of the compound model, enabling likelihood calculation or using it as a generative model. To find an appropriate statistical model, we obtained empirical amplification distributions from experimental data generated with several UMI-based protocols: CEL-seq2, Drop-seq, MARS-seq, and SCRB-seq [13], as well as Smart-seq3 [32] and Smart-seq3xpress [33]. Together, we analyzed 106.3 million UMIs from 856 cells.

**Fig. 5:**
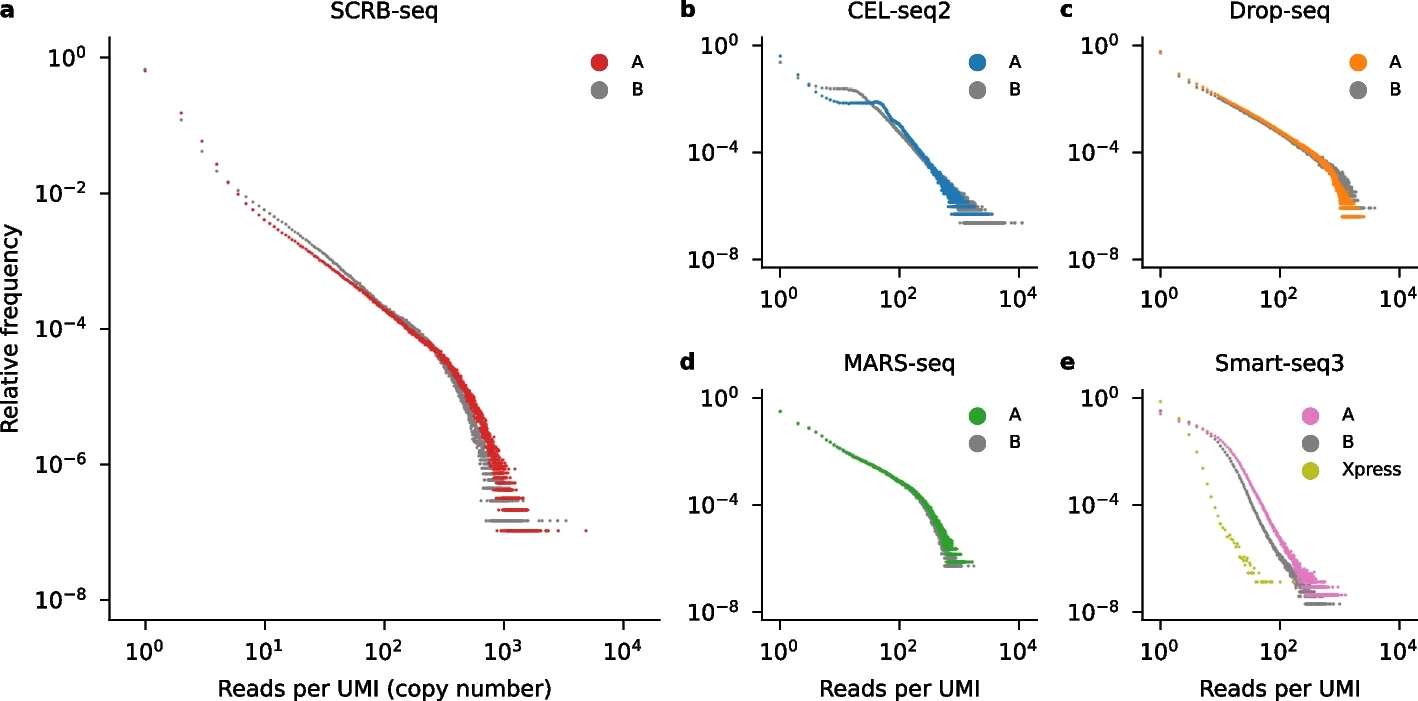
Observed amplification distributions follow a similar shape across protocols. Each panel shows a distribution of UMI copy numbers for a given UMI protocol. **a**–**d** SCRB-seq, CEL-seq2, Drop-seq, and MARS-seq data [13]. For each protocol two identical runs were performed (A and B). **e** Smart-seq3 protocols. Data from a single-end experiment (A), a paired-end experiment (B) [32], and a Smart-seq3 Xpress experiment [33]

For each UMI barcode, we computed the number of times it occurred in the sequenced reads (its copy number). The normalized histogram of these copy numbers provided an empirical characterization of the amplification distribution Z (Fig. 5). Across all sequencing protocols, the copy number histograms in log-log coordinates showed a characteristic elbow shape: Higher copy numbers were less frequent, and the distribution followed two separate decreasing trends in two ranges of copy numbers. This shape can be described by a broken power law, i.e., two separate power laws for low and high copy numbers. The exact shape of the distribution differed between sequencing protocols, leading to different values of mean and variance (Table 1). These values are influenced by how deeply a sample is sequenced and how many cycles of amplification are employed: e.g., the Smart-seq3 Xpress dataset was sequenced shallower than the other two Smart-seq3 datasets (ca. 94000 vs ca. 572000–806000 reads per cell) and with fewer PCR cycles, leading to substantially lower $\mathbb {E}[Z]$ values.

**Table 1:** Key statistics of the observed amplification distribution across protocols. Top part: Each row in the table corresponds to one of the datasets presented in Fig. 5. The number of UMIs shows how many observed copy numbers $z_i$ were used to compute the statistics for that dataset. Bottom row: The Smart-seq2 dataset analyzed in Fig. 4, where UMIs were not observed

Protocol	Run	Cells	UMIs	$\boldsymbol{\mathbb {E}[Z]}$	$\boldsymbol{\mathbb{F}\mathbb{F}[Z]}$	$\boldsymbol{\alpha }_{\boldsymbol{Z}}$	$\boldsymbol{\max (z}_{\boldsymbol{i}}\boldsymbol{)}$
CEL-seq2	A	34	$2\,140\,365$	27.2	107.5	134.8	3476
CEL-seq2	B	37	$4\,303\,956$	24.4	199.8	224.2	11092
Drop-seq	A	42	$2\,506\,244$	29.6	284.2	313.8	2463
Drop-seq	B	34	$1\,272\,895$	31.1	414.1	445.2	3718
MARS-seq	A	29	$1\,342\,232$	24.1	132.1	156.2	1624
MARS-seq	B	36	$1\,903\,673$	20.9	107.0	128.0	1719
SCRB-seq	A	39	$9\,429\,371$	9.7	218.8	228.5	4840
SCRB-seq	B	45	$6\,800\,371$	9.4	159.3	168.7	3276
Smart-seq3	A	145	$21\,549\,849$	5.4	8.0	13.4	1216
Smart-seq3	B	319	$48\,073\,893$	3.8	4.5	8.3	989
Smart-seq3 Xpress		96	$6\,940\,889$	1.3	0.3	1.6	173
Smart-seq2		23822	—	—	—	—	—

To study how stable the amplification distribution was across cells in the same sample, we computed per-cell estimates of $\mathbb {E}[Z]$ and $\alpha _Z = \mathbb {E}[Z] + \mathbb{F}\mathbb{F}[Z]$. The estimates showed some variability across cells (Additional File 1: Supplementary Figure S11), but it was small enough that our simplifying assumption of the shared amplification parameters seems justified in practice. For some protocols, in particular Smart-seq3, the per-cell estimates of $\alpha _Z$ were correlated with the total number of read counts per cell (Additional File 1: Supplementary Figure S12), in agreement to what we saw earlier based on the Smart-seq2 data (Additional File 1: Supplementary Figure S6).

Note that for all protocols, the empirical distribution of copy numbers was monotonically decreasing, meaning that z=1 was the most likely copy number, despite many cycles of amplification. This may seem counter-intuitive, but previous work [34] showed that a mechanistic model of the amplification process followed by Poisson sampling at the sequencing stage can give rise to similar copy number histograms with power law behaviour.

**Fig. 6:**
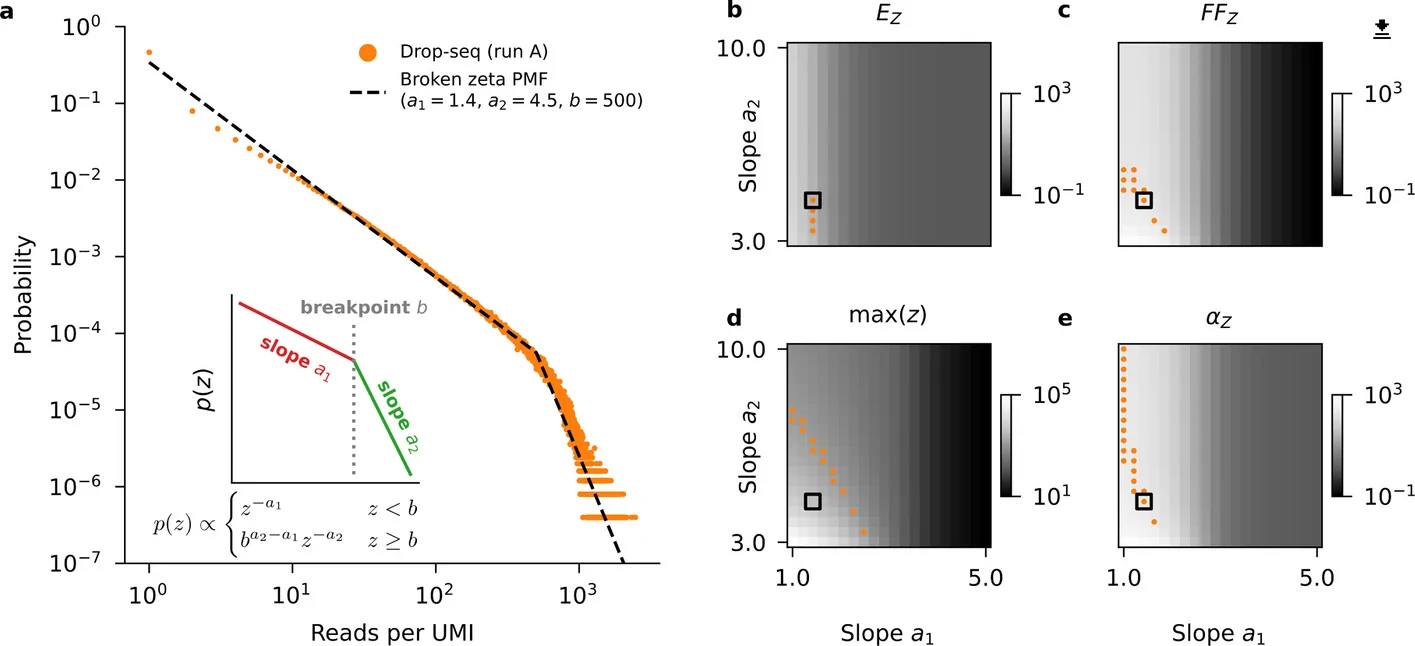
Broken zeta model can fit observed amplification distribution. **a** Observed amplification distribution for Drop-seq (same as in Fig. 5c) (orange dots) and the PMF of a broken zeta model (black line). The inset illustrates the parameters of the broken zeta model. **b** The heatmap shows how the two slope parameters affect the mean of the broken zeta distribution. For each combination of $a_1$ and $a_2$, we sampled from a broken zeta model with these slope parameters and fixed b=500. We used the same sample size as in the observed data in (**a**). The orange dots highlight the simulations yielding the sample mean close the observed mean ($\pm 10\%$). The black square shows the simulation corresponding to the fit shown in (**a**). **c**–**e** Same as (**b**), but showing the sample Fano factor, sample maximum, and sample $\alpha _Z$

As copy numbers are positive integers, we modeled the distribution of copy numbers *z* with a discrete broken power law. The discrete probability distribution with the mass function following a power law is called zeta distribution. For the broken power law, we adopted the term *broken zeta distribution*, which we define as having the following probability mass function (PMF):

15 $$\begin{aligned} p(z) \propto \left\{ \begin{array} {ll} z^{-a_1} & z<b \\ b^{a_2-a_1}z^{-a_2} & z \ge b \\ \end{array}\right. , \end{aligned}$$

where $a_1>0$ and $a_2>0$ are negative slopes of the PMF in log-log coordinates, and $b\in \mathbb {N}$ is the breakpoint between the two slopes (Fig. 6a, inset). We could choose the values for these three parameters such that the broken zeta distribution approximately matched the observed copy number histograms. For example, we obtained a good match for the Drop-seq protocol using $a_1=1.4$, $a_2=4.5$, and b=500 (Fig. 6a).

The fitted model could reproduce several key statistics of the experimental data, such as the mean, the variance, and the Fano factor (Fig. 6b–e). However, the broken zeta distribution produced sample maxima that were larger than empirically observed maxima (given the same sample size) (Fig. 6d). This is a limitation of the broken zeta model as it tends to allocate non-zero probability mass to very high copy numbers that are not observed in practice. A more flexible model that limits the probability of very large copy numbers could potentially fit the data even better, but we considered the broken zeta distribution sufficient for our purposes.

### Compound NB model with broken zeta amplification captures trends in read-count data

**Fig. 7:**
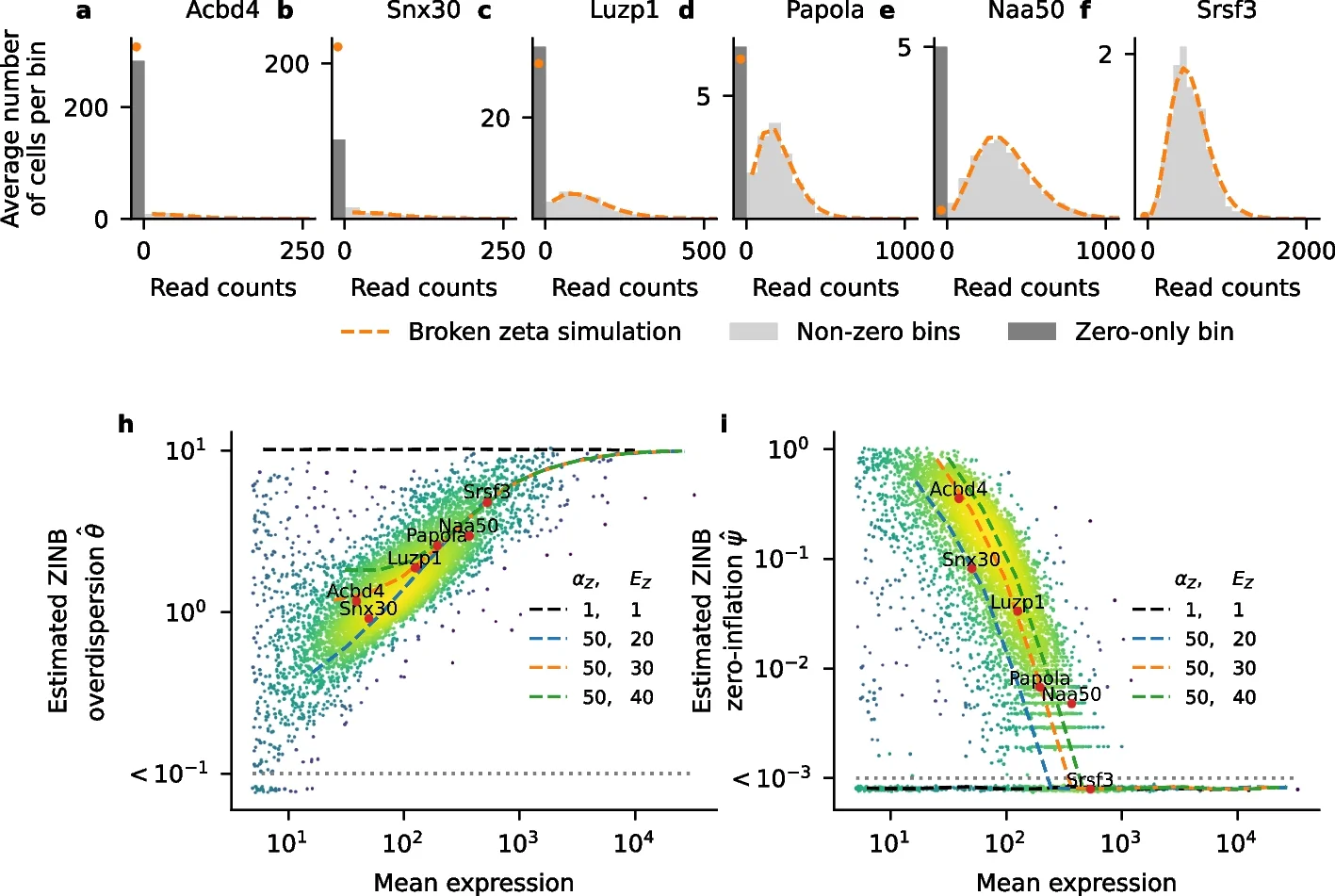
Broken zeta compound model simulations reproduce trends in read counts. Using a homogeneous subset (n=1049) of the mouse cortex data (from Fig. 2), we fitted a zero-inflated negative binomial (ZINB) distribution to each gene individually. We also used the broken zeta model ($a_1=0.36$, $a_2=5.1$, b=56) to sample read counts with a given mean expression. **a**–**f** Each panel shows the observed read counts for a certain gene (gray histogram) and the histogram of counts sampled from the broken zeta model (orange line). Exact zeros are shown in a separate bin (orange dots, dark-gray bar). To show the zero bin (width 1) and non-zero bins (width ≫1) on the same scale, the *y*-axis shows the average count per bin. Genes are ordered from left to right by mean expression. Note that with higher expression, the fraction of zeros decreases, and the histogram shape changes from a geometric-looking distribution to a Poisson-looking distribution. **h** Estimated ZINB overdispersion parameter $\hat{\theta }$ as a function of mean expression. Each dot is a gene, colored by local density of points. Values $\hat{\theta }< 10^{-1}$ were clipped. Only genes with mean expression ≥5 are shown. Colored lines show $\hat{\theta }$ for samples from four different broken zeta models. For each model, we sampled counts over a range of expression fractions $p_g$ for $10^5$ cells, each with fixed sequencing depth of $n_c=100\,000$. We used overdispersion θ=10 for all genes. Broken zeta parameters: see Table 2. The black line corresponds to a negative binomial distribution (UMI model without amplification). Red dots highlight genes from panels (**a**)–(**f**). **i** Estimated ZINB zero-inflation parameter $\hat{\psi }$ as a function of mean expression. Otherwise same as (**h**); values $\hat{\psi }< 10^{-3}$ were clipped

The compound NB model (Eqs. 5–8) together with the broken zeta amplification distribution (Eq. 15) provides a generative probabilistic model of the read counts in a biologically homogeneous population. To confirm that the model gives rise to realistic data, we used it to sample read counts and compared them to observed read-count histograms in a biologically homogeneous dataset (Fig. 7). We used the same dataset as above in Fig. 2. For the amplification distribution, we used broken zeta parameter settings ($a_1=0.36$, $a_2=5.1$, b=56) that led to an amplification model with $\alpha _Z=50$ and $\mathbb {E}[Z]=30$, as we showed earlier that these values fit the protein-coding genes in this dataset well (Fig. 2).

We found that the empirical count distributions of real genes (Fig. 7a–f, gray) could be well matched by the compound NB model (Fig. 7a–f, orange). Note that there is only one free parameter per gene in our simulation: $p_g$, the fraction of RNA molecules taken up by this gene. The entire mass function is then determined by this single parameter. While the compound model did not fit every example gene perfectly (Fig. 7b,e), it correctly captured the shapes of the distributions. In particular, for low-expression genes, the compound model predicted strong zero inflation and monotonically decreasing probability of non-zero counts (Fig. 7a–b), while predicting a bell-shaped distribution without excess zeros for high expression genes (Fig. 7f).

In order to study these patterns more systematically, we fitted a zero-inflated negative binomial (ZINB) distribution to the count histograms of each gene separately. ZINB models have been used to model read counts before [28], as read-count data commonly exhibit zero-inflation compared to NB [25, 29]. A ZINB distribution has three parameters: the mean μ, the inverse overdispersion θ, and the zero-inflation parameter ψ. Its mass function is simply a negative binomial mass function with additional mass ψ on zero:

16 $$\begin{aligned} p(z) = \left\{ \begin{array}{ll} \psi + (1-\psi ) \cdot p_{\textrm{NB}}(0,\mu ,\theta ) & \text {for } z=0 \\ (1-\psi ) \cdot p_{\textrm{NB}}(z,\mu ,\theta ) & \text {for } z> 0 \end{array}\right. \end{aligned}$$

where $p_{\textrm{NB}}$ is the NB probability mass function (see Methods). The ZINB distribution reduces to the NB distribution when ψ=0. As in NB, the overdispersion parameter θ controls the shape of the distribution: θ=1 corresponds to the geometric distribution with monotonically decreasing *p*(*x*), while higher values of θ result in more Poisson-like bell shapes ($\theta =\infty$ corresponds to the Poisson case).

By fitting the ZINB model, we obtained independent estimates $\hat{\theta }_g$ and $\hat{\psi }_g$ for each gene (Fig. 7h–i). These estimates exhibited the same two patterns illustrated above for single genes. First, with increasing mean expression, genes tended to have a higher $\hat{\theta }_g$, corresponding to smaller variance, and transitioned from a geometric-like to a Poisson-like shape. Second, with increasing mean expression, genes tended to have a lower $\hat{\psi }_g$, corresponding to less pronounced zero inflation.

The ZINB model cannot explain these trends, as all parameters $\theta _g$ and $\psi _g$ can be chosen independently. In contrast, our compound model naturally gives rise to both effects. To demonstrate this, we repeated the ZINB fitting procedure on counts sampled from various compound NB models (see Methods for the broken zeta parameters), and reproduced both observations over a wide range of mean expressions (Fig. 7h–i, colored lines). As expected, the model matching this dataset’s amplification parameters ($\alpha _Z=50$ and $\mathbb {E}[Z]=30$, orange line, cf. Fig. 2) provided the best match to the bulk of the distribution.

As a sanity check, sampling read counts from a NB model without amplification ($\alpha _Z=1$) and fitting ZINB distribution to the resulting samples recovered the original parameters (Fig. 7h–i, black lines): constant overdispersion θ=10 and absent zero inflation ψ=0. This again shows that a NB model without amplification cannot describe the properties of the read-count data. However, our results suggest that it is not necessary to include explicit zero-inflation like in a ZINB model, as it is naturally arising through the compound model.

## Discussion

In this paper, we derived a parsimonious and theoretically grounded statistical model describing scRNA-seq read-count data without UMIs. Furthermore, we showed that our compound model leads to analytic compound Pearson residuals, a fast, simple, and effective normalization approach for non-UMI data.

Despite the popularity of UMI protocols [35], full-length non-UMI protocols such as Smart-seq2 [9] remain relevant as they have higher sensitivity [13, 14] and allow quantification of reads over full transcripts. This makes read-count data indispensable for detection of splicing variants [17] or profiling of complex tissues with rare cell types [15, 16]. The recently developed Smart-seq3/Smart-seq3xpress protocols [32, 33] contain UMIs on the 5’-end reads but do not have UMIs on internal reads, so our treatment remains relevant for Smart-seq3/Smart-seq3xpress as well.

While UMI counts can be modeled by a Poisson or a negative binomial (NB) distribution [5, 6, 12, 21, 24, 29, 36–38], read counts can not [25, 29]. Instead, they are often modeled by a more flexible zero-inflated negative binomial distribution (ZINB) [28, 29, 39–41]. However, this choice leaves unexplained what mechanism causes zero-inflation and why there are relationships between the gene-specific ZINB parameters, such as less zero inflation for higher mean expression (Fig. 7i).

Our compound model answers these questions. We showed that read counts in biologically homogeneous data can be well described by a compound negative binomial distribution, arising from simple statistical assumptions about the amplification and sequencing processes. Furthermore, we showed empirically that the distribution of copy numbers approximately follows a broken zeta distribution. Together, our compound NB model with amplification modeled by broken zeta yields a generative model reproducing zero-inflation and overdispersion patterns similar to what is observed in read-count data. Compared to the ZINB model with three per-gene parameters (Eq. 16), our model contains only one free per-gene parameter (Eqs. 5–8), and the varying zero-inflation and overdispersion naturally emerge as a function of a gene’s mean expression.

We observed that the distribution of copy numbers in UMI-containing data followed a similar shape across various protocols (Fig. 5), implying that this is a general property of scRNA-seq data. We argued that this shape could be described by a broken power law, and hence we modelled it with a broken zeta distribution. This model is phenomenological, but previous work on mechanistic modelling of PCR amplification followed by Poisson sampling showed that these processes can give rise to similar copy number distributions [34]. We note that fitting parameters for power-law-like data is intrinsically difficult: common approaches such as least squares often return unstable estimates due to low-probability events [42], which is why we avoided automatic parameter fitting. Instead, we qualitatively showed that the broken zeta model can give rise to realistic read-count distributions.

Our compound NB model did not describe all genes perfectly: we found that a subset of genes, mostly pseudogenes, did not follow the compound model, but rather behaved as if they were not amplified (Figs. 2 and 4). Similar bimodal patterns in gene variance have been observed in previous works (e.g., [13, 43]). Pseudogenes are copies of functional genes that contain a mutation making the copy dysfunctional. We can only speculate about the reason causing pseudogene read counts to have less variance: they may behave differently during amplification or sequencing, or perhaps their counts are an artifact of the alignment algorithm (all datasets we analyzed used STAR [44]). In practice, such pseudogenes have less variance than expected under the compound NB model, so they will be filtered out by the gene selection step in our suggested workflow (Fig. 4a).

On the practical side, we used the compound NB model to derive a fast and theory-based normalization procedure for read counts: compound Pearson residuals. They constitute an extension of the UMI Pearson residuals normalization, that has proven to be effective for gene selection and normalization of UMI data [5, 8]. We showed that compound Pearson residuals work well for processing complex read-count datasets, leading to a biologically meaningful gene selection and embeddings. Importantly, we also showed that normalization and gene selection using the non-compound UMI Pearson residuals leads to suboptimal results on read-count data, underscoring the importance of an adequate statistical model.

Compound Pearson residuals only require to set the $\alpha _Z$ parameter of the amplification distribution. Whereas $\alpha _Z$ can be observed directly from the copy number distribution for UMI-containing data (i.e., reads per UMI, Table 1), it is unknown in a Smart-seq2 experiment. We introduced an estimation procedure to infer $\alpha _Z$ directly from data (Additional File 1: Supplementary Figure S2, Eq. 29) and validated it using simulations. At the same time, the results we obtained with compound Pearson residuals did not strongly depend on the exact value of $\alpha _Z\in [10,100]$, and $\alpha _Z=50$ worked well as a default.

Typical approaches to read-count data normalization consist of scaling read counts by a size factor to account for sequencing depth (CPM: counts per million) and sometimes gene length (TPM: transcripts per million [45], or RPKM: reads per kilobase per million [46]), followed by a log-transform [1, 47, 48]. Various methods have been suggested to estimate the required size factors, going beyond CPM/TPM/RPKM [49]: via spike-ins [43, 50], cell pooling [51], housekeeping genes [47], separate scaling for groups of genes [52], or a Bayesian approach [53]. However, all of these methods depend on the log-transform for variance stabilization, which is inherently limited [4] and fails to fully stabilize the variance [3]. In contrast, our compound Pearson residuals use the mean-variance relationship that follows from simple statistical assumptions, and the resulting residuals are variance-stabilized by design and do not require any explicit normalization by gene length.

Two existing methods aim to transform read counts so that their distribution matches the distribution of UMI counts, and one can then apply standard UMI methods for further processing. Census [19] linearly scales the read counts within each cell to set the mode of the count distribution to 1, while qUMI [20] performs quantile normalization within each cell to transform the entire distribution to the typical shape of within-cell UMI counts. In both cases, the transformations are heuristics not based on any generative statistical model, and the transformed data still require UMI-specific normalization. In contrast, our compound Pearson residuals perform necessary normalization directly on the read counts. In practice, we observed that qUMI and Census perform worse than compound Pearson residuals on simulated data, but can lead to qualitatively comparable normalization results on real data. In any case, in contrast to the qUMI and Census heuristics, our method follows from an explicit statistical model that offers theoretical insights into the data generation process underlying read counts. For example, as described above, our model captures previously unexplained patterns in the zero-inflation and overdispersion of read-count data.

One limitation of our model is that it assumes that the amplification distribution is the same for all genes and cells and uses the single amplification parameter $\alpha _Z$ shared by all cells. This is not the case in Census and qUMI, which both use cell-specific adjustments. Reassuringly, we did not observe strong cell-to-cell variability in $\alpha _Z$ estimates (Additional File 1: Supplementary Figures S6, S11), and furthermore saw that the normalization results did not strongly depend on $\alpha _Z$, provided it was in a reasonable range (Additional File 1: Supplementary Figure S9).

## Conclusion

In summary, we show that the compound NB distribution is the appropriate statistical model for read-count data, naturally giving rise to compound Pearson residuals as an effective, convenient and theoretically motivated way of data preprocessing.

## Methods

### Datasets and preprocessing

Our example read-count dataset throughout this paper is the mouse brain dataset from Tasic et al. [15], GEO accession GSE115746 [54]. It contains cells from the primary visual cortex (VISp) and the anterior lateral motor area (ALM), and was sequenced with Smart-seq2. We downloaded the data for both areas from https://portal.brain-map.org/atlases-and-data/rnaseq/mouse-v1-and-alm-smart-seq and used only the exonic counts and applied the same cell filtering as the original authors [15], leading to 23822 cells and 42776 genes with at least one count. We used the Python package mygene to query the mygene.info database [55] for gene type annotations (type_of_gene field) with the Entrez gene identifiers. We queried BioMart to obtain transcript lengths for all Ensemble mouse genes (database *GRCm39*, column ‘Transcript length (including UTRs and CDS)’ [56]).

From these data, we assembled a biologically homogeneous subset by selecting only cells from one of the largest neuronal clusters (named *VISp Penk Col27a1* by the original authors, 1049 cells, 33914 detected genes). These data were used without further filtering in Figs. 2 and 7 and Additional File 1: Supplementary Figures S1 and S3.

To assemble a heterogeneous dataset, we took the full dataset but filtered out genes that were detected in less than 5 cells as in previous work on UMI Pearson residuals [5, 8], leading to 23822 cells and 38510 genes.

To study copy number distributions across protocols, we used the following UMI datasets:

- Mouse embryonic stem cells profiled by CEL-seq2, Drop-seq, MARS-seq, and SCRB-seq [13]; GEO accession GSE75790 [57];
- Mouse fibroblasts profiled with Smart-seq3 paired-end [58]; accession E-MTAB-10148 [59], sample plate2;
- Mouse fibroblasts profiled with Smart-seq3 single-end [32]; accession E-MTAB-8735 [60], sample Smartseq3.Fibroblasts.smFISH;
- HEK293 cells profiled with Smart-seq3Xpress [33]; accession E-MTAB-11467 [61].

For UMI deduplication, we used Hamming distance correction with a threshold of 1. See Table 1 for numbers of cells and UMIs per dataset. The reads-per-UMI tables are available at https://zenodo.org/record/8172702 [62].

We used scanpy 1.9.0 [30] and anndata 0.8.0 [63] for all scRNA data handling in Python 3.8.10, along with sklearn 1.0.2 [64], numpy 1.21.5 [65], and matplotlib 3.5.1 [66].

### Simulation study

To generate a realistic validation dataset with ground truth marker genes *(Simulation I)*, we simulated read counts based on the Smart-Seq2 read counts from mouse neocortex [15] $X_{cg}$ as follows. For each gene *g*, we computed the average expression fractions $p_g=\sum \nolimits _c X_{cg} / \sum \nolimits _{cg} X_{cg}$. For a set *G* of 14 well-known brain cell type marker genes [31] and each of the 133 clusters in the original data, we computed the within-cluster fraction $p_{ig}$, where *i* is the cluster index. For each cell *c*, we computed its total read counts $n_c=\sum \nolimits _g X_{cg}$ and assumed total UMI counts per cell $n_{c}^{\textrm{UMI}} = n_c / 100$. We then generated UMI counts as $k_{cg}\sim\mathrm{NB}(\mu_{cg}^\mathrm{UMI},\theta=100)$, where

17 $$\begin{aligned} \mu _{cg}^{\textrm{UMI}} = \left\{ \begin{array}{ll} n_{c}^{\textrm{UMI}} \cdot p_{i(c)g} & \text {for } g \in G \\ n_{c}^{\textrm{UMI}} \cdot p_g & \text {for } g \not \in G \\ \end{array}\right. , \end{aligned}$$

where *i*(*c*) denotes the cluster assignment of cell *c*. In words, only the marker genes from *G* were allowed to differ between clusters. We then simulated the amplification of each UMI by drawing copy numbers from the shifted geometric distribution $z_i \sim \mathrm Z=\textrm{Geom}_+(\mu =100)$, which corresponds to amplification with $\mathbb {E}[Z]=100$ and $\alpha _Z=199$ (see below). We finally summed the copy numbers for each gene and cell to obtain read counts $X_{cg} = \sum \nolimits _{i=1}^{k_{cg}}{z_i}$ (Eq. 5). We then filtered out genes with less than 5 cells. In order to be able to make comparisons to Census and qUMI, we further subset the genes to only keep genes for which gene-length information was available (as Census and qUMI require this information for TPM normalization, see below). In the end, we were left with 21321 genes.

To obtain a second validation dataset with a richer cluster structure and ground-truth cell types *(Simulation II)*, we used the same simulation setup as above, but allowed all genes to have cluster-specific fractions, i.e.,

18 $$\begin{aligned} \mu _{cg}^{\textrm{UMI}} = n_{c}^{\textrm{UMI}} \cdot p_{i(c)g}. \end{aligned}$$

After filtering as above, *Simulation II* we were left with 21397 genes.

Both simulations generated copy numbers $z_i$ from the shifted geometric distribution $Z=\textrm{Geom}_+(\mu =100)$, which is equivalent to $Z=\textrm{NB}(\mu =99,\theta =1) + 1$, with $z_i\in \mathbb N_+$ being positive integers. The variance of the negative binomial is equal to $99+99^2/1=9900$ and $\mathbb {E}[Z]=100$, so the Fano factor is 99, leading to $\alpha _Z=\mathbb {E}[Z]+\mathbb{F}\mathbb{F}[Z]=199$.

### Mathematical details of the compound negative binomial model

We use the term *compound Poisson/NB distribution* to describe a discrete random variable that is constructed as a sum over a random number of i.i.d. terms. A compound model has an inner and an outer distribution: The inner distribution generates the i.i.d. summation terms (Eq. 6), while the outer distribution governs the number of terms to be summed (Eq. 7). This setup is sometimes called *stopped-sum* distribution [67]. When the outer distribution is the Poisson distribution, the compound model is known as compound Poisson [68], stuttering Poisson [69, 70], or generalized Poisson [71] distribution.

Note that the term *compound distribution* can also have a different meaning: for example, the work on qUMI normalization [20] used the term ‘compound Poisson model’ to describe a Poisson model with rate parameter λ governed by another distribution.

The expectation of a compound random variable $X = \sum \nolimits _{i=1}^k{z_i}$ with inner distribution $z_i \sim Z$ and outer distribution k∼K can be obtained as follows:

19 $$\begin{aligned} E[X] = \mathbb {E}_K\left[ \mathbb {E}_X[X\mid K]\right] = \mathbb {E}_K\left[ \mathbb {E}_X[z_1 + z_2 + \dots + z_k \mid K]\right] = \mathbb {E}_K\left[ k \cdot \mathbb {E}_X[Z]\right] = \mathbb {E}[K] \cdot \mathbb {E}[Z]. \end{aligned}$$

The variance can be computed similarly:

20 $$\begin{aligned} \text {Var}[X]&= \mathbb {E}_K\left[ \text {Var}[X\mid K]\right] + \text {Var}_K\left[ \mathbb {E}[X\mid K]\right] \end{aligned}$$

21 $$\begin{aligned}&= \mathbb {E}_K\left[ \text {Var}[z_1 + z_2 + \dots + z_k \mid K]\right] + \text {Var}_K\left[ \mathbb {E}[z_1 + z_2 + \dots + z_k \mid K]\right] \end{aligned}$$

22 $$\begin{aligned}&= \mathbb {E}_K\left[ k \cdot \text {Var}[Z]\right] + \text {Var}_K\left[ k \cdot \mathbb {E}[Z]\right] \end{aligned}$$

23 $$\begin{aligned}&= \mathbb {E}[K] \cdot \text {Var}[Z] + \text {Var}[K]\cdot \mathbb {E}[Z]^2. \end{aligned}$$

Together, this leads to the following mean-variance relationship:

24 $$\begin{aligned} \text {Var}[X] = \mathbb {E}[X] \cdot \frac{\text {Var}[Z]}{\mathbb {E}[Z]} + \frac{\text {Var}[K]}{\mathbb {E}[K]^2}\cdot \mathbb {E}[X]^2. \end{aligned}$$

We use the negative binomial (NB) distribution as outer distribution in our compound model. The probability mass function for the NB distribution can be parametrized in several different ways. We use

25 $$\begin{aligned} p_{\textrm{NB}}(k,\mu ,\theta ) = \frac{\Gamma (k+\theta )}{k!\, \Gamma (\theta )}\left( \frac{\mu }{\mu +\theta }\right) ^k \left( \frac{\theta }{\theta +\mu }\right) ^\theta , \end{aligned}$$

where μ is the mean and θ is the overdispersion parameter. The variance is then given by $\text {Var}[K]= \mathbb {E}[K] + \mathbb {E}[K]^2/\theta = \mu +\mu ^2/\theta$.

Plugging the mean-variance relationship of *K* into the mean-variance relationship of *X*, we finally get

26 $$\begin{aligned} \text {Var}[X]&= \mathbb {E}[X] \cdot \frac{\text {Var}[Z]}{\mathbb {E}[Z]} + \frac{\mathbb {E}[K] + \mathbb {E}[K]^2/\theta }{\mathbb {E}[K]^2} \cdot \mathbb {E}[X]^2 \end{aligned}$$

27 $$\begin{aligned}&= \mathbb {E}[X] \cdot \frac{\text {Var}[Z]}{\mathbb {E}[Z]} + \frac{\mathbb {E}[X]^2}{\mathbb {E}[K]} + \frac{\mathbb {E}[X]^2}{\theta } \end{aligned}$$

28 $$\begin{aligned}&= \mathbb {E}[X] \cdot \underbrace{\left( \frac{\text {Var}[Z]}{\mathbb {E}[Z]} + \mathbb {E}[Z] \right) }_{\alpha _Z} + \frac{E[X]^2}{\theta }, \end{aligned}$$

where we used the fact that $\mathbb {E}[X] = \mathbb {E}[Z] \cdot \mathbb {E}[K]$. The relationship in Eq. 28 yields the lines shown in Fig. 2a.

From here we can obtain the relationship between the mean of *X* and the Fano factor of *X*:

29 $$\begin{aligned} \mathbb{F}\mathbb{F}[X] = \frac{\text {Var}[X]}{\mathbb {E}[X]} = \alpha _Z + \frac{\mathbb {E}[X]}{\theta }, \end{aligned}$$

shown as lines in Fig. 2b. For $E[X]\ll \theta$, this reduces to $\mathbb{F}\mathbb{F}[X] \approx \alpha _Z$.

To derive the relationship between the mean of *X* and the fraction of zero counts, we note that the inner distribution in our model is strictly positive (z≥1). Any zero count X=0 must thus originate from a k=0 from the outer NB distribution. As a result, we can derive the fraction of zero counts from the NB probability mass function (Eq. 25):

30 $$\begin{aligned} P(X=0) = p_{\textrm{NB}}(K=0) = \left( \frac{\theta }{\theta + \mathbb {E}[K]}\right) ^\theta = \left( \frac{\theta }{\theta + \mathbb {E}[X]/\mathbb {E}[Z]}\right) ^\theta . \end{aligned}$$

This relationship is shown as lines in Fig. 2c. For $\theta \rightarrow \infty$, this converges to the Poisson case

31 $$\begin{aligned} P(X=0) = e^{-\mathbb {E}[K]} = e^{-\mathbb {E}[X]/\mathbb {E}[Z]}. \end{aligned}$$

In this case, the relationship between mean expression and the log-fraction of zeros is linear:

32 $$\begin{aligned} \log P(X=0) = -\frac{1}{\mathbb {E}[Z]} \cdot \mathbb {E}[X]. \end{aligned}$$

Note that the intercept is zero, and -1/E[Z] is the slope. Consequently, the value of *E*[*Z*] can be estimated by a linear regression without an intercept (Additional File 1: Supplementary Figures S2–S7).

### Compound Pearson residuals

For gene selection with compound Pearson residuals, we computed $\hat{\mathbb {E}}[X_{cg}]$ from the filtered read-count matrix *X* (Eq. 10) and then obtained residuals using Eq. 13. We selected 3000 highly variable genes (HVGs) with the highest residual variance. To normalize, we then subset the raw count data matrix to the HVGs, and computed compound Pearson residuals again on that subset, and used these re-computed residuals for further analysis (consistent with our previous work [8]). Using the residuals computed from the full data matrix and subsetting them to HVGs led to very similar results.

Unless otherwise stated, we used $\alpha _Z=50$ and θ=100 for computing residuals, and clipped residuals to $\sqrt{n}$ where *n* is the number of cells [5] (see our previous work [8] for a motivation for this heuristic).

### Census counts and qUMIs

We obtained both Census counts and qUMIs via their official R implementations using R 4.1.3. To obtain Census counts, we used bioconductor-monocle 2.22.0 [19, 72]. To obtain qUMIs, we used quminorm 0.1.0 from http://github.com/willtownes/quminorm/ [20]. As both methods expect TPMs as input, we subset the [15] data to the 27841 genes for which length annotations were available (see above), and computed TPM from read counts $X_{cg}$ and gene lengths $l_g$ (in kilobase) as

33 $$\begin{aligned} \mathrm{TPM}_{cg} = \frac{X_{cg}/l_g}{\sum \nolimits _g{X_{cg}/l_g}} \cdot 1\,000\,000 \end{aligned}.$$

Running Census on the full matrix was very slow (${>}24$ h), so we split the TPM matrix into batches of 1000 cells. This substantially sped up the computation. Filtering Census counts and qUMIs for genes with at least 5 cells yielded 23822 cells and 25248 genes.

### t-SNE visualizations

As basis for all t-SNE embeddings, we computed the first 1000 principal components (PCs) of the HVGs residuals with sklearn 1.0.2 [64]. For all t-SNE embeddings, we used openTSNE 0.6.0 [73] with default settings unless otherwise stated. To ensure comparability between the t-SNE embeddings in Additional File 1: Supplementary Figure S9, we used the first two PCs of the HVG residuals computed with $\alpha _Z=10$ (panel C) as shared initialization after scaling them with openTSNE.initialization.rescale().

To visualize expression strength of a given gene across the t-SNE map (Fig. 4c), we used square-root-transformed depth-normalized counts

34 $$\begin{aligned} S_{cg} = \sqrt{m \cdot \frac{X_{cg}}{\sum \nolimits _i{X_{ci}}}}, \end{aligned}$$

where *m* is the median row sum of *X*.

### Fitting zero-inflated negative binomial (ZINB) models to single genes

To obtain per-gene estimates of overdispersion $\hat{\theta }_g$ and zero inflation $\hat{\psi }_g$ in the absence of biological variability, we fitted a ZINB model to the raw read counts of each gene in the *VISp Penk Col27a1* cluster (n=1049 cells). We used the ZeroInflatedNegativeBinomialP.fit_regularized() function from statsmodels 0.13.2 [74] with default parameters. Only the 11549 genes with within-cluster mean expression ${>}5$ were included because low-expression genes suffered from unstable parameter estimates. All genes with fitting warnings (n=2064), fitting errors (n=22) or invalid resulting estimates $\hat{\psi }_g>1$ (n=2359) were excluded, such that 7104 genes with valid converged estimates remained for further analysis and are shown in Fig. 7h–i.

We applied the same fitting procedure to the simulated read counts shown as lines in Fig. 7 (see below for simulation details). Here, 62 out of 100 simulated genes had a mean expression ${>}5$ and were used for fitting. 5 of them resulted in invalid $\hat{\psi }_g>1$ values and were excluded, such that 57 simulated genes remained for plotting and analysis.

### Sampling copy numbers from the broken zeta model to simulate compound model read counts

The broken zeta model we describe in Eq. 15 is the discrete version of the broken power law. A continuous probability distribution with probability density following a power law is called the Pareto distribution. Its discrete analogue is known under various names including Riemann zeta distribution (or simply zeta distribution), discrete Pareto distribution, and Zipf distribution [67]. We therefore refer to the discrete broken power law distribution as *broken zeta distribution*. While continuous broken power law distributions are commonly used in astrophysics [75], we are not aware of any prior use of discrete broken power law distributions for statistical modeling.

For the simulations in Figs. 6 and 7, we sampled copy numbers from a given broken zeta distribution. For that, we computed the approximate PMF for a limited support $z\in \{1, 2, \dots , 10^5\}$ with Eq. 15, and normalized the resulting probabilities to sum to 1. This way we did not need to compute the normalization constant in Eq. 15.

For the simulations of the Drop-seq copy number distribution in Fig. 6b–e we used $n=2\,506\,244$ samples per parameter set, which is the number of UMIs in the Drop-seq A dataset. We extended the support until $10^6$ for this particular simulation, because we observed $\max (z_i) \approx 10^5$ for some of the more extreme parameter combinations.

In order to sample realistic read counts from four compound models with different combinations of mean copy number $\mathbb {E}[Z]$ and $\alpha _Z$ (Fig. 7), we used a grid search over the broken zeta parameters $a_1$, $a_2$, and *b* to find parameter combinations that best matched the required values. Table 2 lists the parameters and amplification statistics of the chosen models. Across all parameter sets shown in Fig. 7, we observed $\max (z_i)=7\,179$ which was far below the end of the support.

**Table 2:** Broken zeta parameters used for compound model read-count simulations. Each row in the table corresponds to one of the four compound model simulations shown in Fig. 7. Observed values refer to the obtained sample mean and sample variance (n≈2 billion, see text for simulation details). The model with $\alpha _Z=1$ corresponds to the UMI case with constant copy number $z_i=1$, and thus has no broken zeta parameters

$\boldsymbol{\mathbb {E}[Z]}$		$\boldsymbol{\alpha }_{\boldsymbol{Z}}$				
Target	Observed	Target	Observed	$\boldsymbol{a}_{\boldsymbol{1}}$	$\boldsymbol{a}_{\boldsymbol{2}}$	*b*
1	1.0	1	1.0	–	–	–
20	20.1	50	50.4	0.96	15.1	91
30	30.2	50	51.4	0.36	5.1	56
40	37.9	50	50.5	0.01	17.6	71

We first sampled unique sequenced molecules $k_{cg}$ from a negative binomial with $k_{cg} \sim \mathrm{NB}(p_g n_c, \theta )$ for 25 genes over a log-spaced range of expression fractions $p_g \in [10^{-8}, 10^{-1}]$. We did this for $10^5$ cells, each with fixed sequencing depth of $n_c=10^5$. We used overdispersion parameter θ=10 for all genes. This led to a total of $\sum {k_{cg}}=2\,047\,196\,087$ simulated UMIs. Then, for each of them, we sampled a copy number $z_i$ from the broken zeta model as described above, and summed over copy numbers for the same cell and gene to obtain a read count $X_{cg} = \sum \nolimits _{i=1}^{k_{cg}} z_i$. We used the same set of simulated UMIs for the four compound model simulations shown in Fig. 7 and Table 2.

## Supplementary Information

Additional file 1: Supplementary Table S1. Supplementary Figures S1–S12.

Additional file 2. Review history.

## Data Availability

The datasets (GSE115746 [54], GSE75790 [57], E-MTAB-10148 [59], E-MTAB-8735 [60], E-MTAB-11467 [61], reads-per-UMI tables [62]) generated and/or analysed during the current study are publicly available as described in Methods (Datasets and preprocessing section). All analysis code is available under a MIT license at https://github.com/berenslab/read-normalization [76], and is archived in the Zenodo repository https://zenodo.org/doi/10.5281/zenodo.12806891 [77].
