# Supplementary material for: Compound models and Pearson residuals for single-cell RNA-seq data without UMIs
Source: Genome Biol. 2026 Jun 27;27:294. doi: 10.1186/s13059-026-04161-4 (PMC13595543; doi:10.1186/s13059-026-04161-4)
Supplement: Supplementary file 1 [file 13059_2026_4161_MOESM1_ESM.pdf]

Additional File 1:
Supplementary Table S1, Supplementary Figures S1–S12

Sample size	1 000	5 000	10 000	20 000	50 000	100 000
`Scanpy`	0.2 s	1.5 s	2.4 s	4.2 s	12 s	25 s
Compound Pearson residuals	0.7 s	2.8 s	5.4 s	11 s	29 s	62 s
qUMI	3.7 s	11 s	21 s	50 s	159 s	285 s
Census	1 089 s	4 895 s	11 622 s	27 480 s	53 067 s	92 637 s

Table S1: Runtime experiment. Runtimes for datasets of various sample sizes constructed by randomly sampling cells from the Tasic et al. [15] dataset. As qUMI and Census require gene-length information, in this experiment we only used the 25 533 genes for which this information was available. The runtime experiment was performed on a computing cluster with a Xeon E5-2630 v4 CPU (2.2 GHz) and 503 GB RAM.

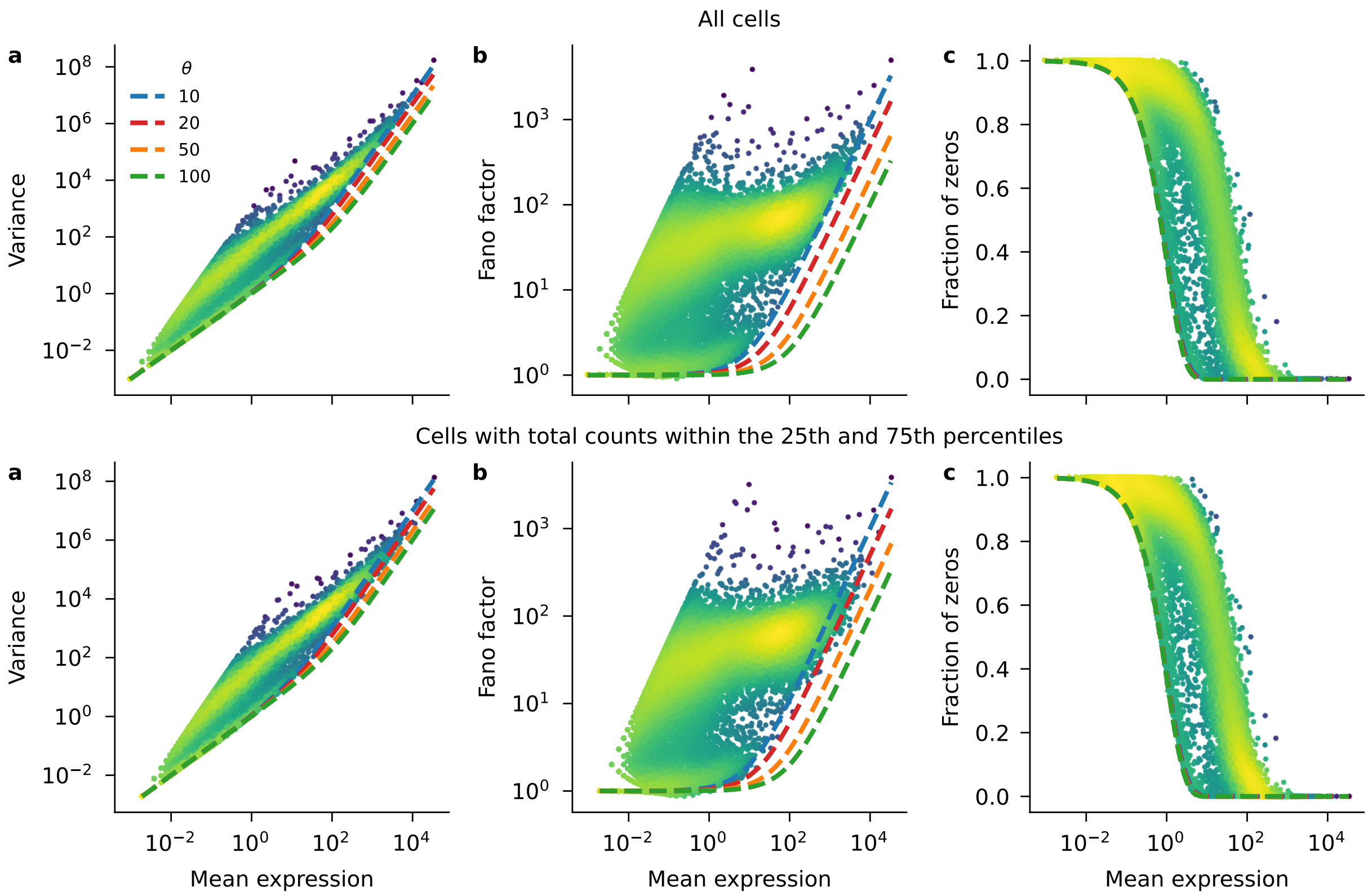

Figure S1: Sequencing depth variation increases the apparent overdispersion. Both rows are a reproduction of Figure 2, but showing pure NB models ($\alpha_Z = 1$) that differ in their overdispersion parameter θ. **a–c:** Plots based on all 1 049 cells as shown in Figure 2 with total counts per cell ranging from ∼680 000 (1st percentile) to ∼2.84 million (99th percentile). Note that the NB model with $\theta = 10$ fits the boundary of the data distributions. **d–f:** Plots based on a subset of the 523 cells with total counts within the 25th and 75th percentiles (from ∼1.54 million to ∼1.90 million reads). Now the NB model with $\theta = 20$ fits the boundary of the data distributions better than with $\theta = 10$. In other words, overdispersion is smaller when controlling for sequencing depth variation.

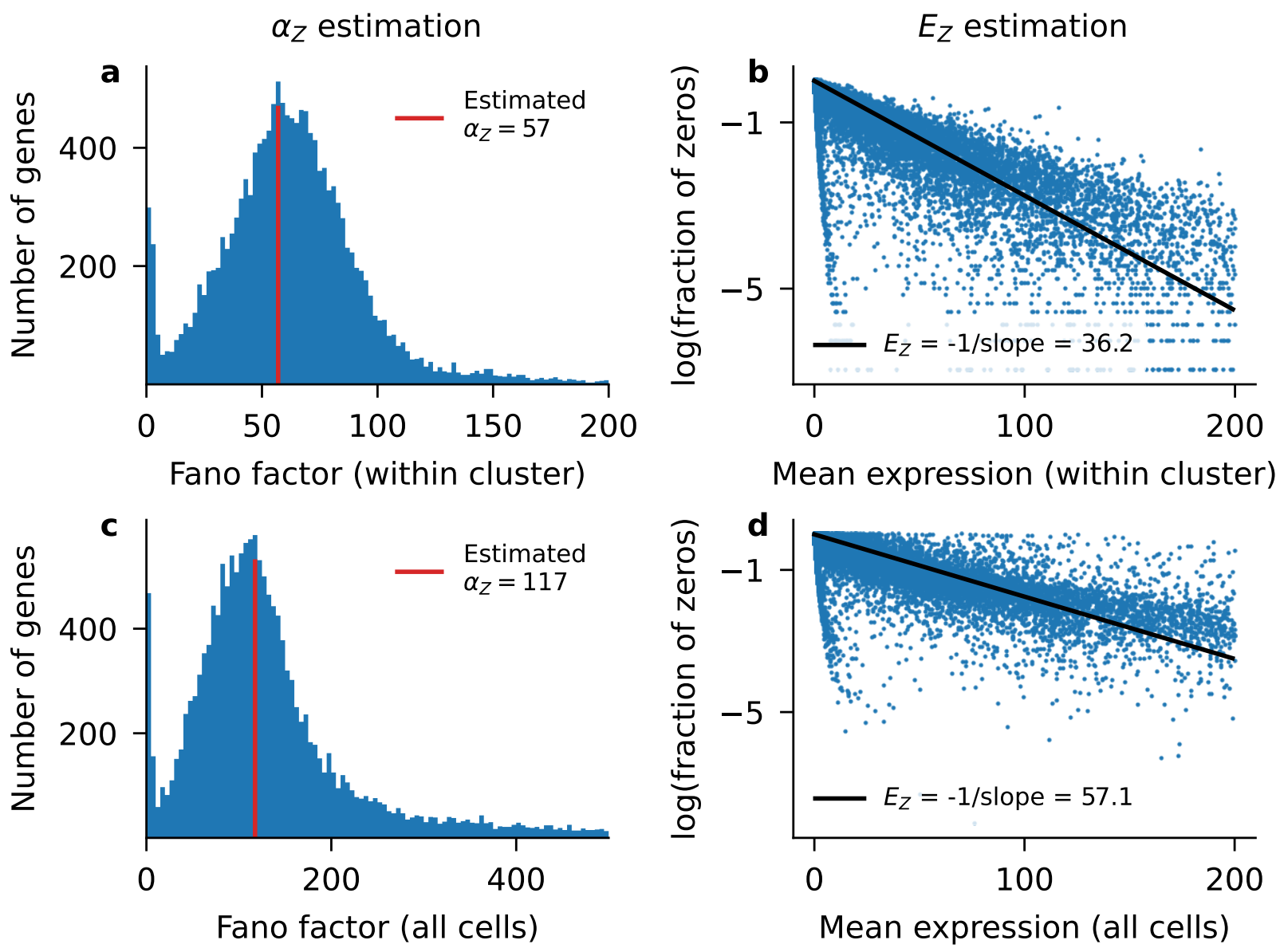

Figure S2: Estimating compound model parameters from real data. Top row: Estimation based only on cells from one example cluster (*L6 IT VISp Penk Col27a1*) used in Figure 2. Bottom row: Estimating parameters from all cells. **a, c:** Fano factor histograms over all genes with mean expression > 1. The red line indicates the mode of the histogram, which is the estimate of α_Z. **b, d:** The relationship between the mean expression and the logarithm of the fraction of zeros for all genes with mean expression in $[1, 200]$ range. Each dot is a gene. The black line is a linear fit without intercept, and its slope is the negative inverse of the estimate of $\mathbb{E}[Z]$.

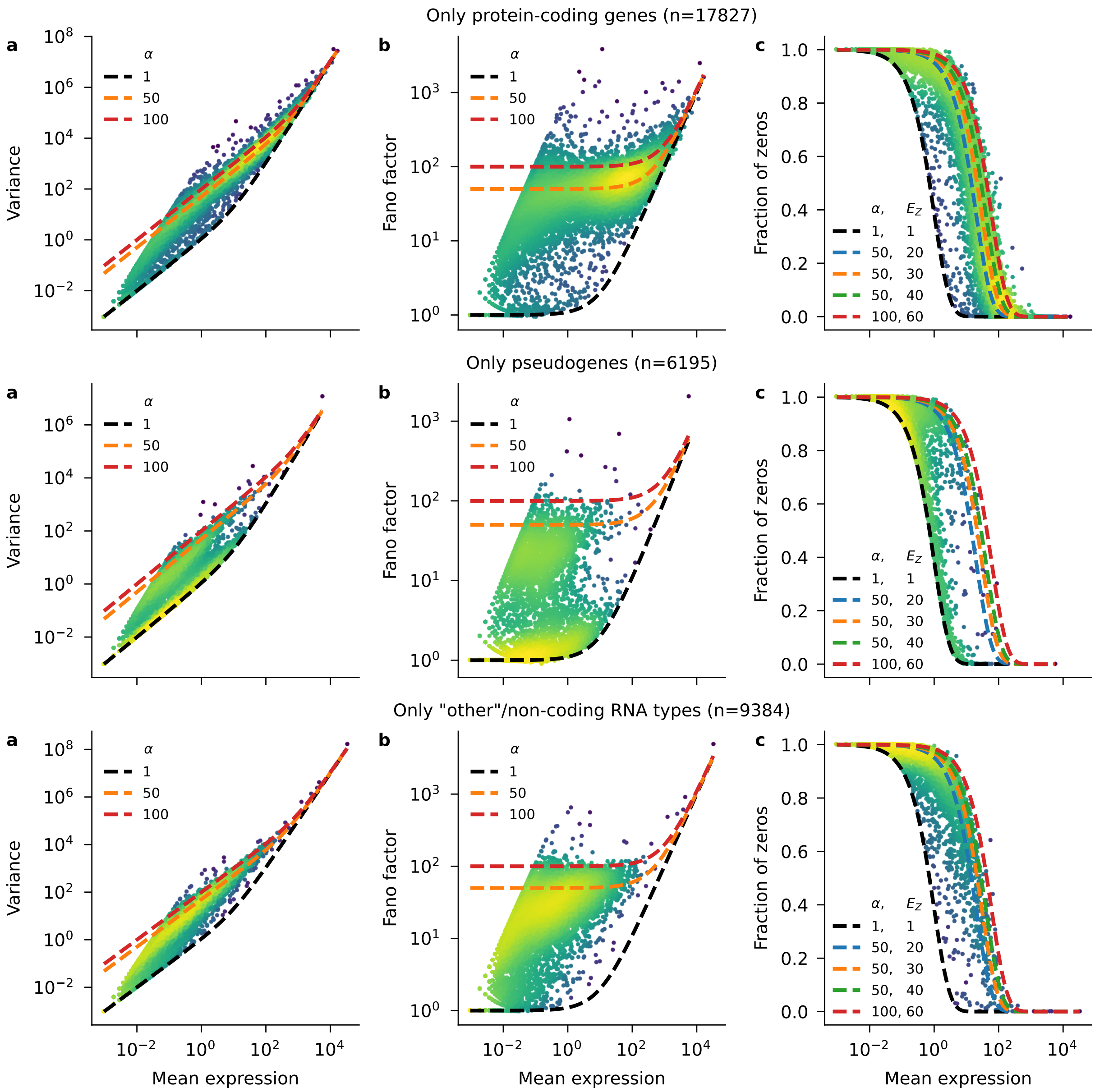

Figure S3: Non-amplified genes are mostly pseudogenes. Each row is a reproduction of Figure 2, but showing only genes from a certain category as obtained by the `mygene.info` annotation service. 508 genes without available annotation are not shown. **a–c:** Protein-coding genes. **d–f:** Pseudogenes. Many of them appear 'non-amplified' and do not follow the compound model, but rather the UMI model without amplification (black). **g–i:** Other, non-coding RNA species. Note that the bulk of these low-expression genes did not follow the compound model either.

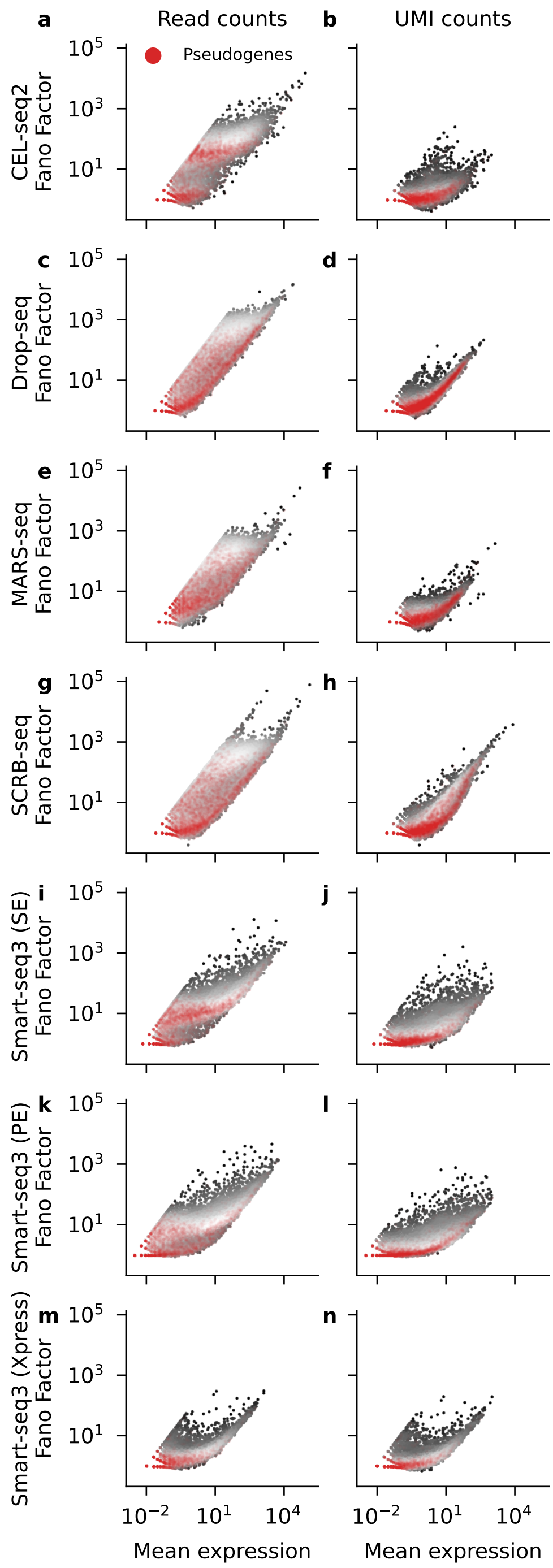

Figure S4: Pseudogenes have low Fano factor of read counts across protocols. Each panel shows the relationship between the mean and the Fano factor for all genes in each dataset. Higher density of dots is shown in brighter gray. Red dots with transparency show pseudogenes. Each row of panels shows a homogeneous dataset sequenced with a different protocol. All left-column panels are based on read counts, all right-column panels are based on UMI counts from the same dataset. Same data as in Figure 5. **a–h**: Mouse embryonic stem cells sequenced with various UMI protocols [13]. For all protocols, only run A is shown. **i–l:** Smart-seq3 data from mouse fibroblasts [32]. SE: Single-end run. PE: Paired-end run. **m–n:** Smart-seq3 Xpress data from HEK293 cells [33].

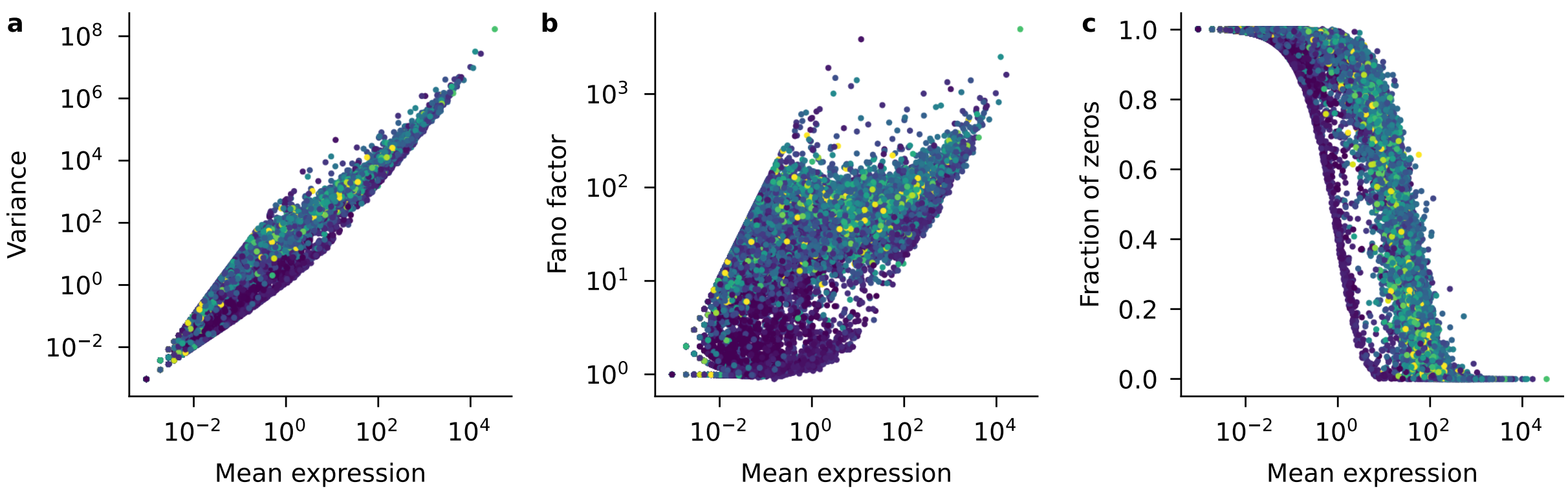

Figure S5: Pseudogenes have lower maximum transcript lengths. Reproduction of Figure 2, but showing each gene colored by the maximum length across all of its transcripts present in the `Ensembl` mouse gene database. 10 846 genes without transcript length annotation are not shown. Maximum lengths were clipped to the 98th percentile (9 832 bp) before plotting.

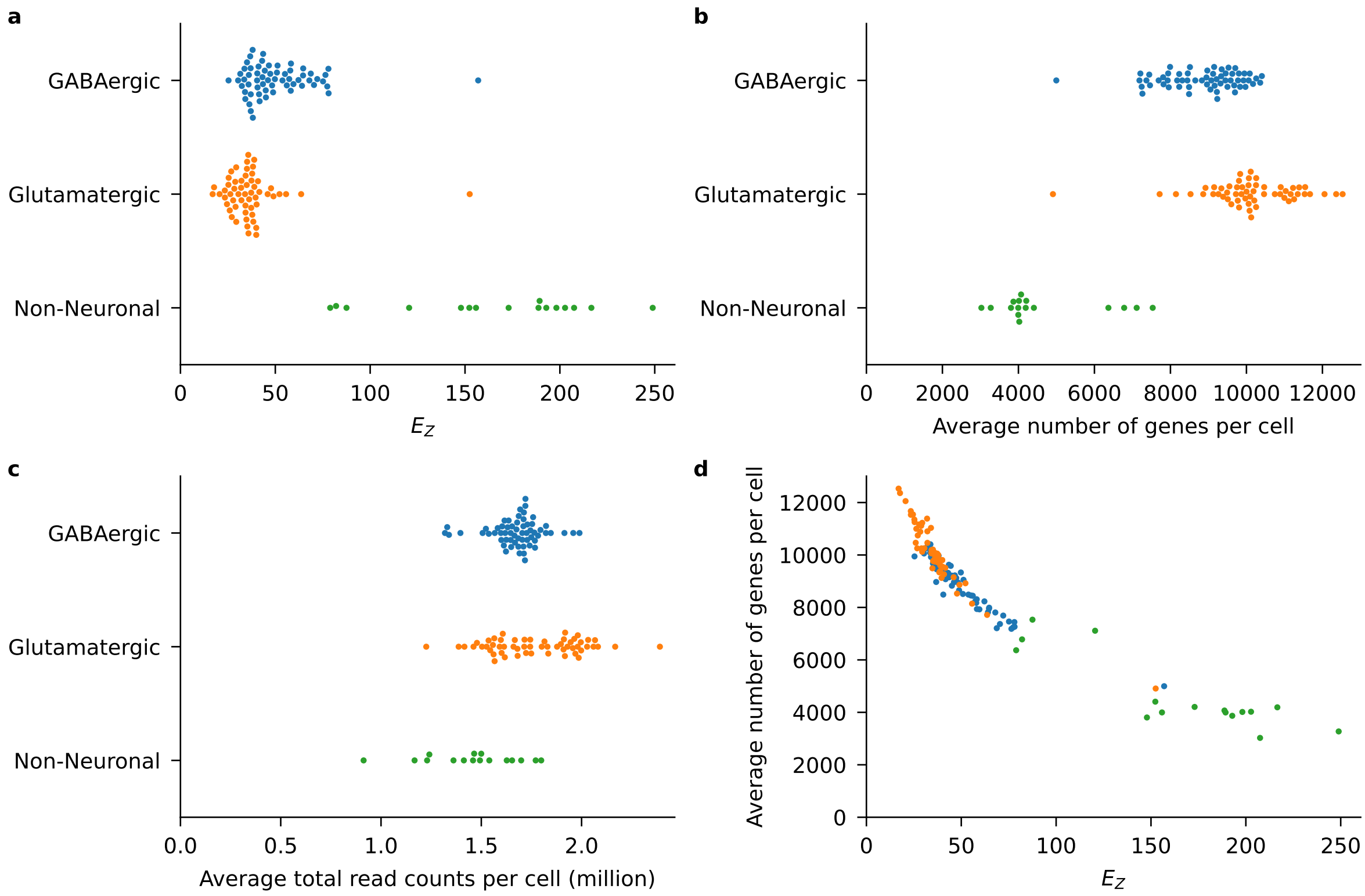

Figure S6: Estimating compound model parameters from real data for each cell type. a: The estimates of $\mathbb{E}[Z]$ obtained as in Figure S2b for each cell type separately. The two outlying neuronal cell types with high $\mathbb{E}[Z]$ are *Meis2 Adamts19* and *CR Lhx5*. **b:** The average number of detected genes per cell type. Note the negative correlation with panel a. **c:** The average number of total read counts per cell, per cell type. All cell types get roughly the same number of reads because the amount of amplified cDNA to be sequenced is experimentally controlled for each cell. See Extended Data Figure 4 in the original paper [15] for the same histograms as here in panels b–c for each cell type. **d:** Strong negative correlation between the number of detected genes and the estimate $\mathbb{E}[Z]$. Cells with fewer genes get more reads per molecule because the total number of reads stays approximately the same.

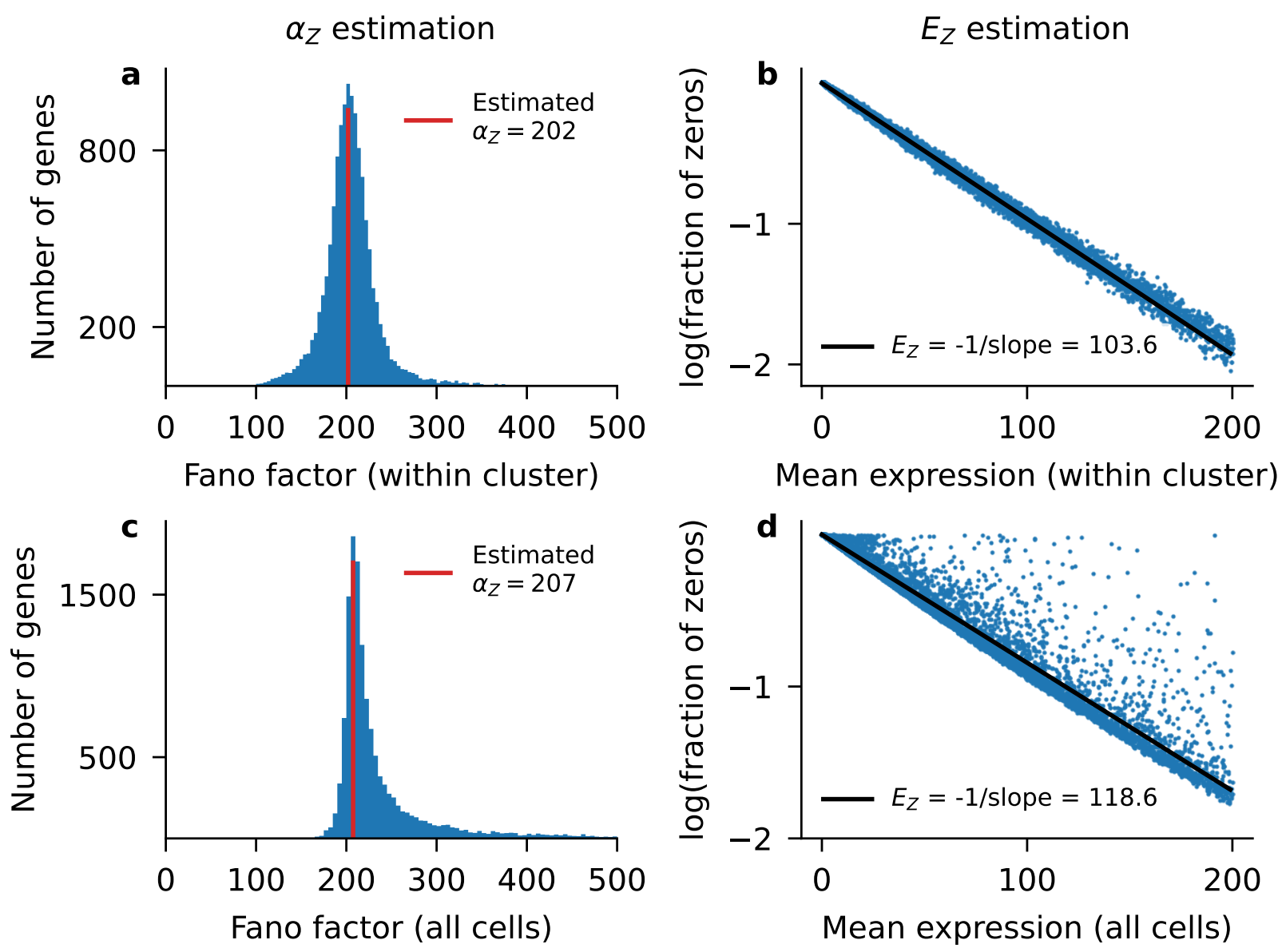

Figure S7: Estimating compound model parameters from simulated data. Same as Figure S2, but based on data from *Simulation II*, where true $\alpha_Z = 199$ and true $\mathbb{E}[Z] = 100$. Both parameter values are recovered well by the estimation procedure.

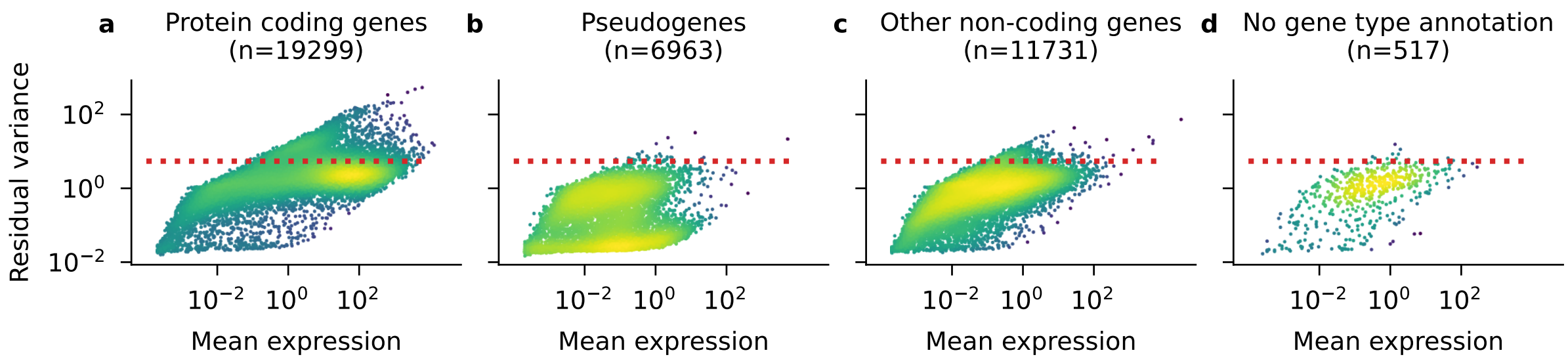

Figure S8: Genes with residual variance $\ll 1$ are mostly pseudogenes. Each panel is a reproduction of Figure 4a, showing only a certain category of genes, as in Figure S3. Each dot represents a gene and shows its mean and residual variance in the full mouse visual cortex dataset [15]. Brighter color indicates higher density of points. Red line shows cutoff for selecting 3 000 HVGs among all genes. Gene type annotations taken from the `mygene.info` service. **a:** Protein-coding genes. **b:** Pseudogenes. **c:** Other, non-coding RNA species. **d:** Genes without available annotation.

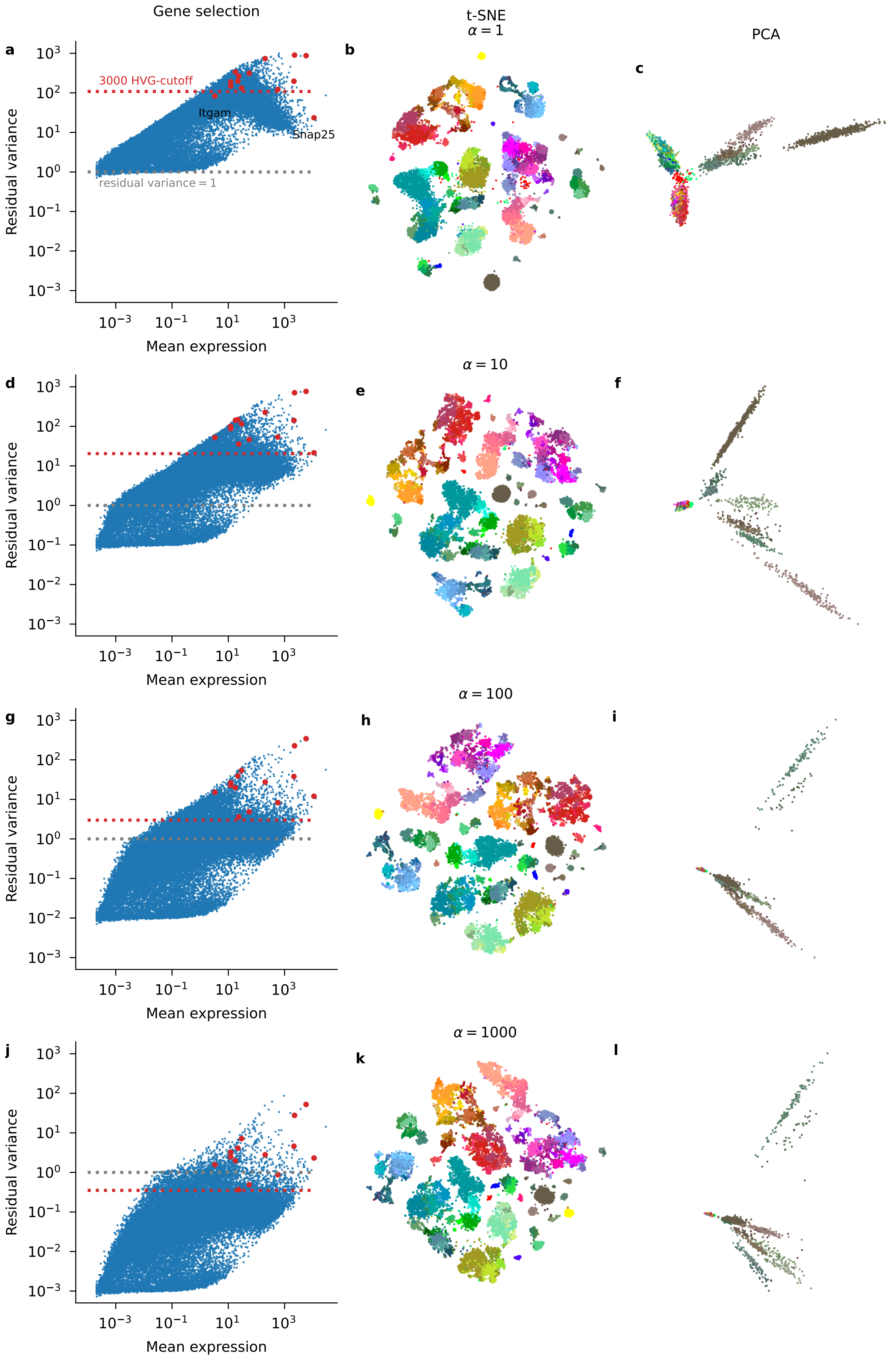

Figure S9: Influence of the amplification parameter α. Each row contains a reproduction of Figure 4a–b for various values of α_Z. The first row corresponds to the NB model without amplification, used for UMI data [8]. Gray line: indicates residual variance = 1, where most non-differentially expressed genes should lie if the model is correct. All t-SNEs used the same shared initialization (see Methods). Right column shows the first two principal components (PCs) of the compound Pearson residuals.

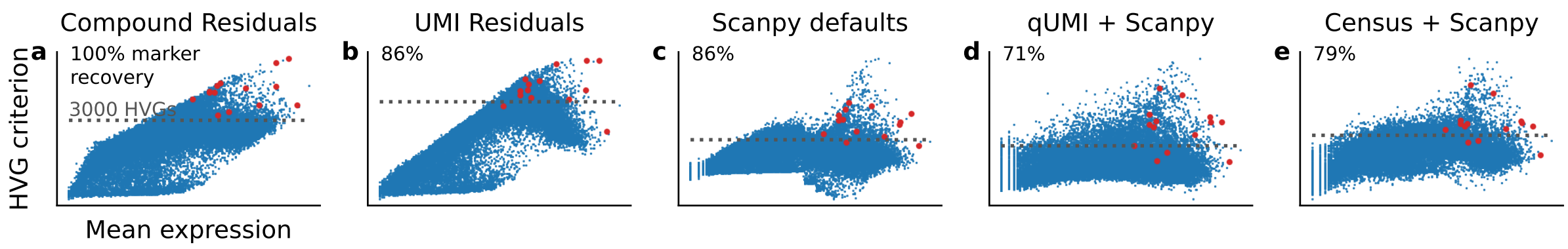

Figure S10: Comparing marker gene selection between compound Pearson residuals and other methods. The same analysis as shown in Figure 3a–e for read counts processed with different methods. The panels plot the log-scaled read-count mean expressions against the HVG selection criterion of the respective method (residual variance on a log scale in a–b and normalized dispersion on a linear scale in c–e). The percentage of recovered marker genes in each panel is the fraction of well-known marker genes among the top 3000 HVGs. Red dots: marker genes (see caption of Figure 4). Dashed lines: cut-off for top 3000 HVGs. **a:** Compound Pearson residuals. **b:** UMI Pearson residuals ($\alpha = 1$). **c:** Standard `Scanpy` normalization. **d:** qUMIs [20] followed by the standard `Scanpy` normalization. **e:** Census counts [19] followed by the standard `Scanpy` normalization.

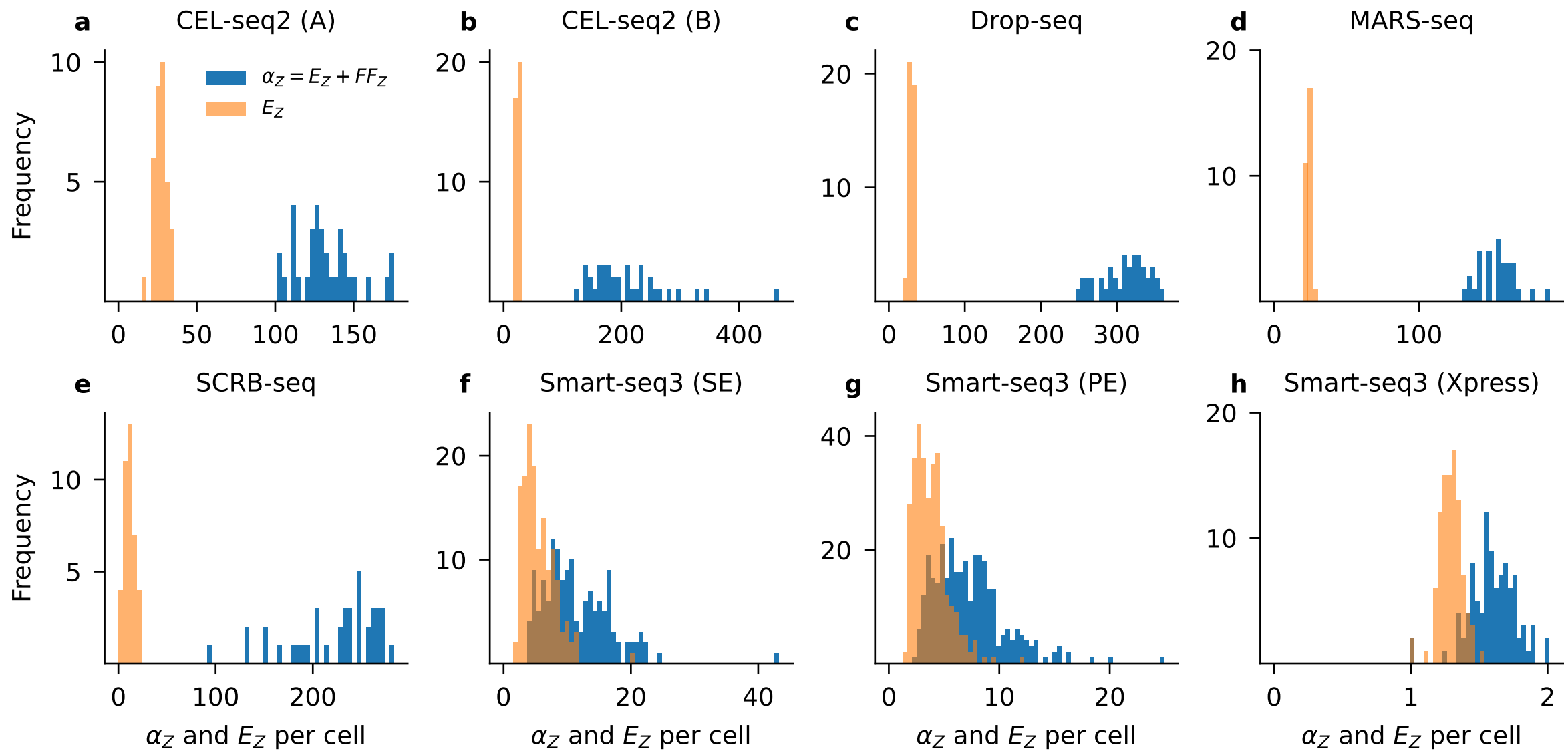

Figure S11: Cell-to-cell variability in α_Z and $\mathbb{E}[Z]$. Each panel shows amplification statistics computed per cell for all sequencing platforms listed in Table 1. Only run A is shown unless otherwise indicated.

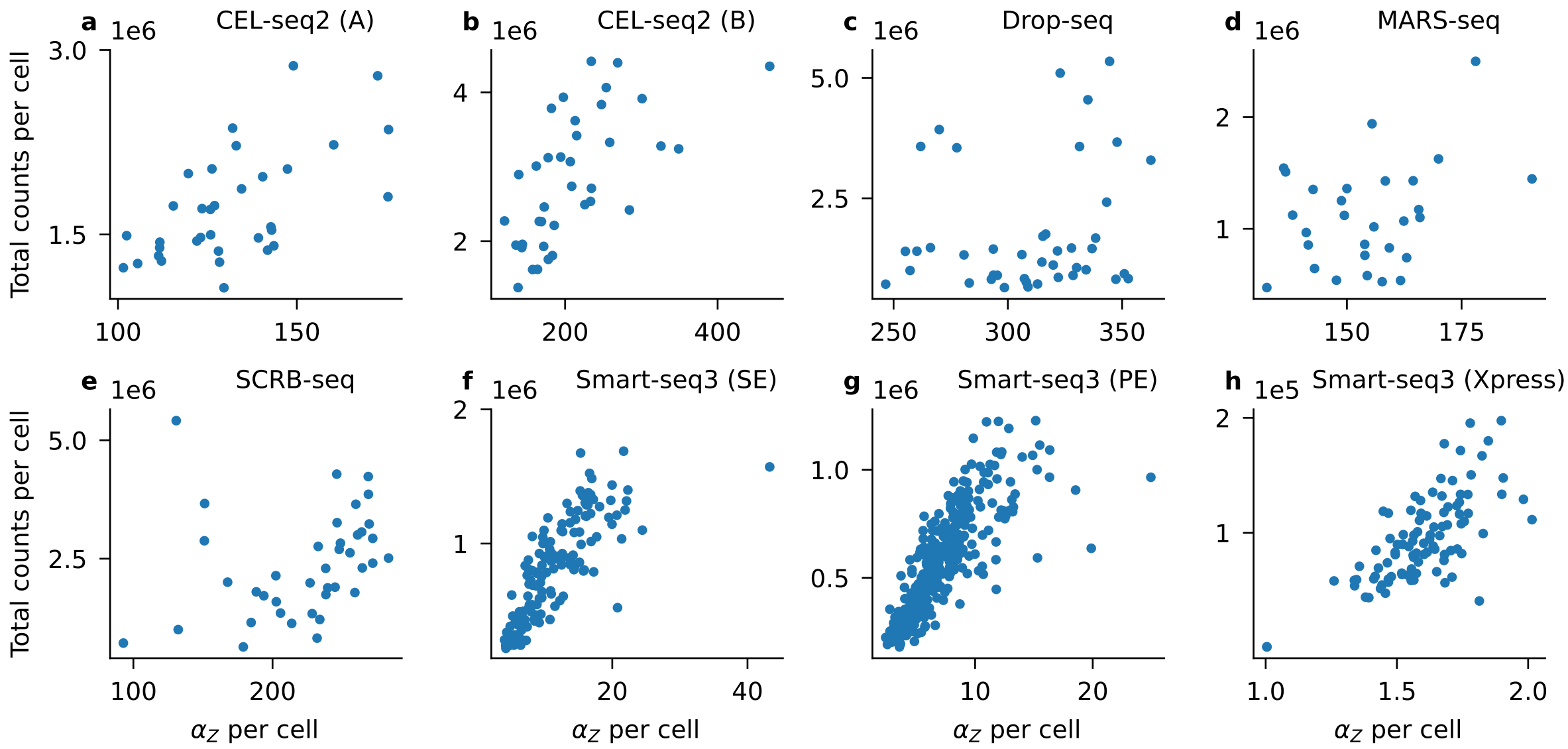

Figure S12: Total read counts are correlated with α_Z. Each panel corresponds to one of the sequencing platforms listed in Table 1; each dot is a cell. Only run A is shown unless otherwise indicated.
