# Supplementary material for: Compound models and Pearson residuals for single-cell RNA-seq data without UMIs
Source: Genome Biol. 2026 Jun 27;27:294. doi: 10.1186/s13059-026-04161-4 (PMC13595543; doi:10.1186/s13059-026-04161-4)
Supplement: Supplementary file 2 [file 13059_2026_4161_MOESM2_ESM.docx]

Review History

**First round of review**

**Reviewer 1**

**Were you able to assess all statistics in the manuscript, including the appropriateness of statistical tests used?**

No

**Were you able to directly test the methods?**

No

**Comments to author:**

The authors do a nice job in providing a mechanistic model for observation frequency of individual genes in complex single cell data. In particular, the authors integrate a simple binomial sample picking regime that describes rarely observed gene counts with an over inflation model that describes excess observation frequencies of highly expressed genes. If a gene is highly prevalent or is very efficiently amplified by PCR, it will saturate the sequencing reads observed in a given cell. Sparsely represented genes , however, are subject to stochastic picking and undersampling in relatively low sequencing depth samples, and form a long tail of genes represented by a single count or by a null count.

This paper does have some limitations:

Although the models presented do a good job at fitting the mean transcript observation rates, the variance of an individual gene is significant, particularly in the case of highly expressed transcripts. This likely reflects the impact of stochastic counting of these highly expressed genes. I don't see how that variation may be reduced without molecular indexing. Is the intent here to define regimes of accurate, low transcript count genes compared to less well-calibrated high transcript count genes?

The authors do benchmark across a variety of UMI based single cell applications, with comparable bimodal fitting characteristics. The fitting parameters, however, vary significantly. Is it intended that these methods are portable across biochemistry platforms? Are these fitting parameters characteristic to individual sample types or are they portable across samples?

The data sets presented are relatively small in terms of cells per sample. Are there concerns regarding scalability of these models to current cell count applications (10k+ cells per sample)?

**Reviewer 2**

**Were you able to assess all statistics in the manuscript, including the appropriateness of statistical tests used?**

No

**Were you able to directly test the methods?**

No

**Comments to author:**

In this manuscript, the authors proposed a compound Pearson residuals based method to normalize single-cell RNA-seq data without UMIs. The validation of this normalization method is only based on downstream analysis, including gene selection and low-dimensional embeddings of Smart-seq2 datasets, which cannot demonstrate the effectiveness of the method. Validation datasets should be involved to assess the effectiveness of the method. Further, comparison with other methods which were also designed for normalization of single-cell RNA-seq data also lack in this manuscript.

**Authors Response**

**Point-by-point responses to the reviewers’ comments:**

We thank the editor and the referees for carefully assessing our manuscript and for the thoughtful suggestions and criticism. The editor summarized the referee reports as follows:

*As you will see from the reports, both referees are broadly favorable and find the work of potential interest, but they raise important issues that we must ask you to address, in the form of a revised manuscript, before we reach a final decision on publication. In particular, Referee 1 comments on the scalability of the approach, and Referee 2 asks for comparisons with existing methods and for a demonstration on a validated dataset.*

We have substantially revised the manuscript in order to address these concerns with the following changes and additional experiments:

1. As requested by Referee 1, the revised manuscript discusses speed and memory requirements of compound Pearson residuals. We describe that our implementation runs in seconds for common dataset sizes, and how it can be scaled to datasets with millions of cells with minor adjustments.
2. As requested by Referee 2, we present a new set of experiments where we simulate validation datasets with known ground truth marker genes and cell type labels, and show that compound Pearson residuals successfully recover this ground truth.
3. As requested by Referee 2, we present additional experiments comparing compound Pearson resid- uals to qUMI and Census normalization, and discuss the conceptual differences between these heuristic approaches and our model-based procedure. We conclude that all three methods perform similar in practice, but only our approach is based on a statistical model and hence gives new insights into the read count data distribution.
4. In response to a comment of Referee 2, we adjusted the title and the abstract to stress that our work is not only contributing a normalization procedure, but also a statistical model that offers new theoretical insights into read count data and the methods researchers use to process them.
5. We have updated the code in our Github repository to include all analysis of this revision and archived the updated version at Zenodo.

For the full list of changes and a point-by-point response to all referee comments please see below.

Overall we believe that these changes strongly improved the manuscript.

**Reviewer 1**

*The authors do a nice job in providing a mechanistic model for observation frequency of indi- vidual genes in complex single cell data. In particular, the authors integrate a simple binomial sample picking regime that describes rarely observed gene counts with an over inflation model that describes excess observation frequencies of highly expressed genes. If a gene is highly prevalent or is very efficiently amplified by PCR, it will saturate the sequencing reads ob- served in a given cell. Sparsely represented genes , however, are subject to stochastic picking and undersampling in relatively low sequencing depth samples, and form a long tail of genes represented by a single count or by a null count. This paper does have some limitations:*

*Although the models presented do a good job at fitting the mean transcript observation rates, the variance of an individual gene is significant, particularly in the case of highly expressed transcripts. This likely reflects the impact of stochastic counting of these highly expressed genes. I don’t see how that variation may be reduced without molecular indexing. Is the intent here to define regimes of accurate, low transcript count genes compared to less well- calibrated high transcript count genes?*

**Q1: Variation between genes and aim of our method.** As the referee correctly points out, not using molecular indexing (i.e. UMIs) will inevitably lead to some additional variability in read count data compared to UMI data. Also, the amount of variability per gene will depend on its average expression. We want to clarify that the purpose of our model is not to reduce this variation, but to predict the expected amount of variation for each gene and cell under the null model of only technical variation. This allows computing residuals, which includes “equalizing” variation between genes with different expression levels (variance stabilization), and thereby allows both low- and high-expression genes to contribute to downstream analysis — but only if they are informative, i.e. contain variation that cannot be explained by the null model.

We have revised the manuscript in various places to stress that this theory-based normalization variance stabilization is the “intent” of our model setup.

*The authors do benchmark across a variety of UMI based single cell applications, with com- parable bimodal fitting characteristics. The fitting parameters, however, vary significantly. Is it intended that these methods are portable across biochemistry platforms? Are these fitting parameters characteristic to individual sample types or are they portable across samples?*

**Q2: Portability across platforms and samples.** As the referee correctly points out, Table 1 shows the amplification parameters E[*Z*] and *α_Z_* vary between platforms, i.e. the amplification distributions differ in their means and variances between platforms. Still, our Figure 4 suggests that amplification distributions share the same functional form of a broken power law across platforms. When we obtained estimates of E[*Z*] and *α_Z_* for multiple runs/samples from the same platform, these estimates were much more similar within than between platforms, i.e. while not perfectly stable, the amplification distribution seems to be similar for multiple runs of the same platform.

What does this mean for the practical application of our compound Pearson residuals normalization method? For experimental non-UMI readcount data, the true *α_Z_* is unknown, as one would need UMIs to directly observe the amplification and compute its statistics. We show in our Supplementary Figure S6 that as long as the amplification parameter *α_Z_* ≫ 1, our method produces reasonable results over a large

range of *α_Z_* values. We therefore assume a value of 50 or 100 is likely to work well across protocols.

Furthermore, under the common assumption that most genes in a sample are common housekeeping genes without differential expression between cell types, one can empirically estimate *α_Z_* for a given dataset by adjusting it such that the majority of genes has residual variance equal to 1. We have extended a paragraph in the discussion on practical recommendations and now explicitly mention this procedure.

*The data sets presented are relatively small in terms of cells per sample. Are there concerns regarding scalability of these models to current cell count applications (10k+ cells per sample)?*

**Q3: Scalability of compound Pearson residuals.** Scalability is an important concern, but in fact is not an issue for our method. While the control datasets we used to study ground truth amplification distributions are indeed very small (most of them *<*100 cells, listed in Table 1, we performed all other experiments on the Tasic et al. (2018) dataset with ca. 23 000 cells. For this dataset, we can compute the full matrix of residuals in ca. 15 seconds on a single CPU. For clarity, we have now added the Tasic et al. (2018) dataset to Table 1.

In general, the computational complexity of compound Pearson residuals is the same as for UMI Pearson residuals. We showed in our previous work Lause et al. (2021) that with advanced implementa- tions such as that by Irizarry (2021), this type of method can be scaled to datasets with millions of cells. However, for economic reasons, full-length read count datasets are typically much smaller than this.

We have added a discussion of runtimes, memory requirements, and advanced implementations to the Results section.

**Reviewer 2**

*In this manuscript, the authors proposed a compound Pearson residuals based method to normalize single-cell RNA-seq data without UMIs.*

**Q1: Scope of the paper.** We want to emphasize that providing a fast and effective framework for non-UMI scRNA-seq data normalization is only one of two main contributions of our paper: the other is the compound model itself. This explicit statistical model of read count data follows from simple assumptions about read count data generation, and sheds light on previously unexplained patterns in read count zero-inflation and overdispersion.

In the revised version, we added some theoretical analysis and edited the manuscript to put more emphasis on the theoretical contributions of our work that underlie the normalization framework we suggest here. Specifically, we have slightly adjusted the title and rewrote the abstract.

We also added Equation 14 that captures the relation between compound- and UMI Pearson residuals, and a discussion of our model assumption to share the amplification parameter *α_Z_* between cells (Results Section 2.6, Supplementary Figures S10 and S11, Discussion).

*The validation of this normalization method is only based on downstream analysis, including gene selection and low-dimensional embeddings of Smart-seq2 datasets, which cannot demon- strate the eflectiveness of the method.*

*Validation datasets should be involved to assess the eflectiveness of the method.*

**Q2: Validation on a ground truth dataset.** As the referee correctly noted, we focused the evaluation of our method on downstream processing on a heterogeneous Smart-seq2 dataset. We believe that the best way to benchmark normalization methods is via downstream tasks, because this is what normalization methods are for. However, we agree that the lack of ground truth cell type annotations and marker genes in real datasets limits such benchmarks.

Therefore, we addressed the referee’s request for validation datasets by adding additional experiments with simulated data (Supplementary Figure S9). These simulations generate realistic read count data for which we know the ground truth class of each cell and whether or not a gene is truly differentially expressed, i.e. a true marker gene. We applied our compound Pearson residuals normalization to the simulated data, and showed that our method recovered both ground truth clusters and marker genes.

Furthemore, using the simulated datasets we showed that our method is robust against misspecifica- tions of the amplification parameter *α_Z_* in a certain range, while UMI Pearson residuals (equivalent to *α_Z_* = 1) lead to undesirable normalization outcomes — underscoring that compound Pearson residuals are a required extension to Pearson residuals-based normalization.

We now present these findings in an additional subsection in the Results, and describe the details of the simulation in a new Methods subsection.

*Further, comparison with other methods which were also designed for normalization of single- cell RNA-seq data also lack in this manuscript.*

**Q3 Comparison with existing read count normalization methods.** We thank the referee for raising this point. We have added comparisons to qUMI (Townes and Irizarry, 2020) and Census (Qiu et al., 2017) normalization to our paper. We observed that qUMI and Census combined with UMI Pearson residuals lead to comparable normalization results as our compound Pearson residuals (Supplementary Figure S7).

However, we want to stress that both qUMI and Census rely on heuristics to recover UMIs from read counts. In contrast, our method follows from an explicit statistical model that offers theoretical insights into the data generation process underlying read counts. For example, as described above, our model captures previously unexplained patterns in the zero-inflation and overdispersion of read count data (Results section 2.7).

Also, our model offers fast (15 s) and theory-based normalization in one single step, while qUMI and Census are slower (qUMI 3 min, Census 6 h) and require additional UMI-specific normalization steps on top.

The revised manuscript presents this comparison in the Results section and discusses the implications in the Discussion section.

**References**

Rafael Irizarry.	R package with methods for small counts stored in a sparse matrix. https://github.com/rafalab/smallcount, 2021.

Jan Lause, Philipp Berens, and Dmitry Kobak. Analytic Pearson residuals for normalization of single-cell RNA-seq UMI data. *Genome Biology*, 22(1):1–20, 2021.

Xiaojie Qiu, Andrew Hill, Jonathan Packer, Dejun Lin, Yi-An Ma, and Cole Trapnell. Single-cell mRNA quantification and differential analysis with Census. *Nature Methods*, 14(3):309–315, 2017.

Bosiljka Tasic, Zizhen Yao, Lucas T Graybuck, Kimberly A Smith, Thuc Nghi Nguyen, Darren Bertag- nolli, Jeff Goldy, Emma Garren, Michael N Economo, Sarada Viswanathan, et al. Shared and distinct transcriptomic cell types across neocortical areas. *Nature*, 563(7729):72–78, 2018.

*F William Townes and Rafael A Irizarry. Quantile normalization of single-cell RNA-seq read counts without unique molecular identifiers. Genome Biology, 21(1):1–17, 2020.*

**Second round of review**

**Reviewer 2**

The authors have added additional experiments and comparisons. However, these experiments are still insufficient to fully assess the method’s effectiveness.
The authors use residual variance maximization to select highly variable genes (HVGs), but it remains unclear whether this method captures all relevant marker genes. A detailed analysis of the overlap among the top 3,000 genes selected by different methods is recommended to assess consistency and identify potential marker genes missed by individual methods.
Quantifying the separation of cell types in the t-SNE embedding, along with consistency with annotated labels, would better demonstrate the advantages of this method. For example, statements such as "the default Scanpy preprocessing showed less local structure" lack a clear definition of what constitutes "local structure," and similarly, "compound Pearson residuals provide a more meaningful representation of the data" does not adequately explain why the representation is considered superior.
Further, a table summarizing runtime and memory usage across multiple datasets would help clarify the computational efficiency of each method.

**Reviewer 3**

The proposed compound negative binomial (NB) model is statistically sound and conceptually interesting. The formulation, which links observed read counts to a compound process involving sampling and amplification, is both novel and well-motivated. The authors demonstrate its validity using UMI-based datasets.

However, I have concerns about the practical application of this model to non-UMI data, which is its intended target. In such cases, the amplification parameter alpha_Z is not estimated from data, but rather either assumed based on prior experience or selected by optimizing the variance of Pearson residuals after normalization. The former approach does not truly require the compound NB model, as it treats alpha_Z as a fixed constant. Also, The latter approach is quite ad hoc and introduces a circular logic, since the amplification distribution should be determined by the sequencing protocol itself, not inferred from the properties of the normalized output. In other words, although the authors propose a principled model, their estimation procedure does not meaningfully make use of it in the non-UMI setting.

From a methodological perspective, a better strategy would be to treat Z (representing the amplification distribution) as a latent variable and estimate it within the model. This would avoid the need for post hoc plug-in values and preserve the internal consistency of the compound NB framework. A Bayesian approach may be well-suited here, in which UMI-based datasets could be used to define informative priors over the amplification distribution that can then be applied to non-UMI data. This would allow the model to leverage available information in a principled way, while maintaining both flexibility and coherence when applied to datasets lacking UMIs.

**Authors Response**

**Point-by-point responses to the reviewers’ comments:**

**Reviewer 2**

*The authors have added additional experiments and comparisons. However, these experiments are still insufficient to fully assess the method’s eflectiveness.*

*The authors use residual variance maximization to select highly variable genes (HVGs), but it* ***remains unclear whether this method captures all relevant marker genes****. A detailed analysis of the overlap among the top 3,000 genes selected by diflerent methods is recommended to assess consistency and identify potential marker genes missed by individual methods.*

***Quantifying the separation of cell types*** *in the t-SNE embedding, along with consistency with annotated labels, would better demonstrate the advantages of this method. For example, statements such as "the default Scanpy preprocessing showed less local structure" lack a clear definition of what constitutes "local structure," and similarly, "compound Pearson residuals provide a more meaningful representation of the data" does not adequately explain why the representation is considered superior.*

We thank the reviewer for their comment. We agree that it is difficult to clearly define representation quality, especially for real data without ground-truth labels. In the case of the Tasic et al. (2018) dataset, the only available cell-type labels were derived by the original authors via clustering, which itself depends on the originally used preprocessing pipeline.

To take your concern seriously, we have thus decided to extend our two simulation experiments and use them for method comparison.

- In *Simulation I*, only a small set 14 marker genes is differentially expressed. In this simulation, a good HVG selection would select the true markers first, as they are the only truly variable genes. We evaluated HVG selection performance by counting the fraction of true markers in the top 14 selected HVGs. Only compound Pearson residuals recovered all true markers, while all other methods performed worse – in particular, qUMI and Census.
- In *Simulation II*, we allowed all genes to vary around the means of the Tasic et al. (2018) clusters, yielding 133 ground-truth cell types. A good normalization method would preserve these cell types for downstream analysis. To quantify this, we computed *k*NN accuracy after HVG selection and PCA, i.e., the fraction of cells for which the majority of their 10 nearest neighbors has the same cell type. Here, compound Pearson residuals were performing similar to the default Scanpy normalization, but again clearly better than the dedicated read-count processing methods qUMI and Census.

We combined these results in a new main figure (Figure 3 in the manuscript), reproduced here as well, and described the results in a separate main text section.

Additionally, we removed the qualitative statements that the reviewer rightly criticized.

Figure 1: **Compound Pearson residuals recover ground truth in simulated data. a–e:** *Sim- ulation I*, in which the expression of only 14 genes varied between simulated cell types. The panels show the relationship between mean expression in the simulated read count data and the HVG selection criterion of the respective method (residual variance in a–b and normalized dispersion in c–e), both on the log scale. Each dot is one of 21 321 simulated gene; red dots correspond to simulated marker genes. **f–j:** *Simulation II*, in which the expression of all 21 397 simulated genes was allowed to vary between simulated cell types. The panels show *t*-SNE embeddings of the data after the respective transformation, followed by HVG selection and PCA. Each dot is a cell, colored by simulated cell type. Numbers in each panel denote the *k*NN accuracy (top) and the balanced *k*NN accuracy (bottom) for cell type prediction in PCA space.

*Further, a table summarizing runtime and memory usage across multiple datasets would help clarify the computational efficiency of each method.*

We thank the reviewer for this suggestion. In a more systematic runtime comparison, we subsam- pled/upsampled the Tasic et al. (2018) dataset to sample sizes from 1 000 to 100 000 (see following table, that we also added as a supplementary table to the manuscript). Compound Pearson residuals had only a minor runtime overhead over the standard Scanpy preprocessing, and were much faster than qUMI and Census.

| sample size | 1 000 | 5 000 | 10 000 | 20 000 | 50 000 | 100 000 |
| --- | --- | --- | --- | --- | --- | --- |
| Scanpy | 0.2 s | 1.5 s | 2.4 s | 4.2 s | 12 s | 25 s |
| Compound Pearson residuals | 0.7 s | 2.8 s | 5.4 s | 11 s | 29 s | 62 s |
| qUMI | 3.7 s | 11 s | 21 s | 50 s | 159 s | 285 s |
| Census | 1 089 s | 4 895 s | 11 622 s | 27 480 s | 53 067 s | 92 637 s |

Table 1: **Runtime experiment.** Runtimes for datasets of various sample sizes constructed by randomly sampling cells from the Tasic et al. (2018) dataset.

**Reviewer 3 (new in this round)**

*The proposed compound negative binomial (NB) model is statistically sound and conceptually interesting. The formulation, which links observed read counts to a compound process involv- ing sampling and amplification, is both novel and well-motivated. The authors demonstrate its validity using UMI-based datasets.*

We thank the Reviewer for this positive assessment.

*However, I have concerns about the practical application of this model to non-UMI data, which is its intended target. In such cases,* ***the amplification parameter*** *α_Z_* ***is not estimated from data****, but rather either assumed based on prior experience or selected by optimizing the variance of Pearson residuals after normalization. The former approach does not truly require the compound NB model, as it treats α_Z_ as a fixed constant. Also, The latter approach is quite ad hoc and introduces a circular logic, since the amplification distribution should be determined by the sequencing protocol itself, not inferred from the properties of the normalized output. In other words, although the authors propose a principled model, their estimation procedure does not meaningfully make use of it in the non-UMI setting.*

*From a methodological perspective, a better strategy would be to* ***treat Z (representing the amplification distribution) as a latent variable and estimate it within the model****. This would avoid the need for post hoc plug-in values and preserve the internal consistency of the compound NB framework. A Bayesian approach may be well-suited here, in which UMI- based datasets could be used to define informative priors over the amplification distribution that can then be applied to non-UMI data. This would allow the model to leverage available information in a principled way, while maintaining both flexibility and coherence when applied to datasets lacking UMIs.*

We thank the reviewer for this thoughtful comment. We note that in the previous version of the manuscript, we were using a qualitative fit to statistics of the raw read counts to determine a good value for *α_Z_* (Figure 2 in the manuscript). However, we agree that this procedure left room for improve- ment.

We therefore devised a more rigorous procedure that follows directly from our model and can estimate both amplification parameters *α_Z_* and E[*Z*]:

- The observed Fano factors can be used directly to estimate the amplification parameter *α_Z_*, because in our model, for genes with low average expression, the Fano factor of the read counts is equal to *α_Z_* (Equation 11 and 29 in the Methods). Based on this relationship, we estimated *α_Z_* from the mode of the Fano factor distribution (see new Supplementary Figure S2, also reproduced below, panels a,c).
- Further, the value of E[*Z*] can be estimated using a linear fit (Equations 31–32 in the Methods) to the relationship between the log-transformed fraction of zeros and the mean expression (panels b,d).

Figure 2: **Estimating compound model parameters from real data.** Top row: Estimation based only on cells from the example cluster (*L6 IT VISp Penk Col27a1* ) in Figure 2. Bottom row: Estimating parameters from all cells. **a, c:** Fano factor histograms over all genes with mean expression *>* 1. The red line indicates the mode of the histogram, which is the estimate for *α_Z_* (see Results/Methods). **b, d:** Mean-log-fraction-of-zeros relationship for all genes with mean expression *>* 1 and *<* 200, i.e., restricting to a range where the fraction of zeros is neither all-zero nor all-one; each gene is a blue dot. The black line is a linear fit without intercept, and its slope is the negative inverse of the estimate for E*_Z_*.

Furthermore, we validated this estimation procedure using *Simulation II* described in our reply to Reviewer 2, where we know the ground-truth *α_Z_* and E[*Z*]. The procedure recovered the true values almost perfectly. See new Supplementary Figure S3, also reproduced below.

Even though this procedure falls short of a full Bayesian procedure that the reviewer had in mind, we believe that our approach is fast, accurate, and sufficient for this problem.

Figure 3: **Estimating compound model parameters from simulated data.** Same as Figure 2, but based on data from Simulation II, where true *α_Z_* = 199 and true E*_Z_* = 100 (see Results/Methods). Both are recovered well by the estimation procedure.

**References**

Bosiljka Tasic, Zizhen Yao, Lucas T Graybuck, Kimberly A Smith, Thuc Nghi Nguyen, Darren Bertag- nolli, Jeff Goldy, Emma Garren, Michael N Economo, Sarada Viswanathan, et al. Shared and distinct transcriptomic cell types across neocortical areas. *Nature*, 563(7729):72–78, 2018.

**Third round of review**

**Response to the editor**

We thank both the editor and the reviewers for the constructive review process and for taking the time to revisit the manuscript one more time.

As requested by the editor, we have now added an additional method comparison that reviewer #2 suggested, which demonstrated that compound Pearson residuals performed better than existing methods for marker gene recovery in real data. This is in line with our simulation results from the last revision.

We have also added one additional supplementary figure (new Figure S6) to further clarify one aspect of our presentation. This new figure is briefly referenced in the main text.

*Many thanks for submitting the revised version of your manuscript entitled ’Compound models and Pearson residuals for single-cell RNA-seq data without UMIs’ to Genome Biology. Please accept my apologies for the delay in getting back to you about it. It has now been seen again by Referee #3 and their comments are accessible below. Once the outstanding editorial concerns are satisfactorily addressed, we would, in principle, be delighted to publish your manuscript.*

We thank the editor for the positive assessment. We have addressed all editorial concerns (i.e., added dataset repository and GitHub citations, updated Declarations section as requested, changed to numbered citation style, and separated the supplementary material into a separate file), and now look forward to the final steps towards publication.

*Previously, Reviewer #2 asked for a comparison of the overlap in marker genes among the top 3,000 HVGs selected by diflerent methods to assess consistency and potential missed markers. We appreciate you added 2 simulations in the current revised manuscript, however it remains unclear how the diflerent methods compare in capturing marker genes. Could you please add this analysis in a further revised version?*

As requested, we have now added a method comparison for how many marker genes are captured among the top 3 000 HVGs, and we present the results in a new Supplementary Figure S10 (also pasted below as Figure R1). We found that compound Pearson residuals captured more well-known markers in the top 3000 HVGs than the other methods. We note that this is well in line with our simulation I (main text Figure 3a–e), where we found that compound Pearson residuals outperformed other methods in recovering the ground truth marker genes.

*Additionally, we have made some minor changes to the title and the abstract of the manuscript, to make them better adhere to Genome Biology’s house style. Please find the edited versions pasted at the foot of this email. We ask that you include these versions in any subsequent submissions of your manuscript.*

Thanks for your edits to the abstract. We have made a few more minor changes for clarity, but still kept the length below 100 words as requested. Regarding the title, we would like to note that our paper is not only about (compound) Pearson residuals, but also about compound model for non-UMI data in general. To emphasize this, we would like to keep our previous title (“Compound models and Pearson residuals for . . . ”) instead of the suggested “Compound Pearson residuals for . . . ” — unless that title violates some formal constraints of Genome Biology.

Figure R1: **Comparing marker gene selection between compound Pearson residuals and other methods.** The same analysis as shown in Figure 3a–e for read counts processed with different methods. The panels plot the log-scaled read-count mean expressions against the HVG selection criterion of the respective method (residual variance on a log scale in a–b and normalized dispersion on a linear scale in c–e) The percentage of recovered marker genes in each panel is the fraction of well-known marker genes among the top 3000 HVGs. Red dots: marker genes (see caption of Figure 4 Dashed lines: cut-off for top 3000 HVGs. **a:** Compound Pearson residuals. **b:** UMI Pearson residuals (*α* = 1). **c:** Standard Scanpy normalization. **d:** qUMIs followed by the standard Scanpy normalization. **e:** Census counts followed by the standard Scanpy normalization.

*Thank you for depositing your code on Github under a GPL3 license, as well as archiving on Zenodo (CC BY 4.0). For clarity you may want to consider assigning the same license to both repositories so that the same ‘clauses’ apply to users regardless of the download location.*

Thanks for pointing us to this inconsistency. We have now changed both licenses to the more permissive MIT license.

*Please provide a reference for your source code in zenodo, and for your github repository where mentioned in the text, and include these in the reference list, as with other references. This should be in the following format: Last, First. [all authors] Title. Github. hyperlink/doi (year of deposition). Last, First. [all authors] Title. Zenodo. hyperlink/doi (year of deposition).*

*We request that you also formally cite the used third-party datasets, when these have persistent identifiers, such as a DOI or accession number, in addition to the URLs of the papers that presented described these data. This is to ensure full credit is given to those who share data with the community. Please formally cite the GEO and E-MTAB datasets, as well as the reads-per-UMI tables on Zenodo. This should be in the following format: Last, First. [all authors] Title. Datasets. Gene Expression Omnibus. hyperlink/doi (year of deposition).*

We have added citations for all datasets and our GitHub and Zenodo code repositories as requested.
